# Mechanisms of different response to ionizing irradiation in isogenic head and neck cancer cell lines

**DOI:** 10.1186/s13014-019-1418-6

**Published:** 2019-11-27

**Authors:** Vesna Todorovic, Ajda Prevc, Martina Niksic Zakelj, Monika Savarin, Andreja Brozic, Blaz Groselj, Primoz Strojan, Maja Cemazar, Gregor Sersa

**Affiliations:** 1 Department of Experimental Oncology, Institute of Oncology Ljubljana, Ljubljana, Slovenia; 2 Department of Cytopathology, Institute of Oncology Ljubljana, Ljubljana, Slovenia; 3 Department of Radiation Oncology, Institute of Oncology Ljubljana, Ljubljana, Slovenia; 4 University of Ljubljana, Faculty of Medicine, Ljubljana, Slovenia; 5 University of Primorska, Faculty of Health Sciences, Izola, Slovenia; 6 University of Ljubljana, Faculty of Health Sciences, Ljubljana, Slovenia

**Keywords:** Head and neck cancer, Squamous cell carcinoma, Radioresistance, Radiotherapy, Cancer recurrence, Chemoresistance, Radiotherapy, Gene expression profiles, DNA damage, DNA repair

## Abstract

**Background:**

Treatment options for recurrent head and neck tumours in the previously irradiated area are limited, including re-irradiation due to radioresistance of the recurrent tumour and previous dose received by surrounding normal tissues. As an in vitro model to study radioresistance mechanisms, isogenic cells with different radiosensitivity can be used. However, they are not readily available. Therefore, our objective was to establish and characterize radioresistant isogenic human pharyngeal squamous carcinoma cells and to evaluate early radiation response in isogenic parental, radioresistant and radiosensitive cells.

**Methods:**

Radioresistant cells were derived from parental FaDu cells by repeated exposure to ionizing radiation. Radiosensitivity of the established isogenic radioresistant FaDu-RR cells was evaluated by clonogenic assay and compared to isogenic parental FaDu and radiosensitive 2A3 cells. Additional phenotypic characterization of these isogenic cells with different radiosensitivity included evaluation of chemosensitivity, cell proliferation, cell cycle, radiation-induced apoptosis, resolution of DNA double-strand breaks, and DNA damage and repair signalling gene expression before and after irradiation.

**Results:**

In the newly established radioresistant cells in response to 5 Gy irradiation, we observed no alteration in cell cycle regulation, but delayed induction and enhanced resolution of DNA double-strand breaks, lower induction of apoptosis, and pronounced over-expression of DNA damage signalling genes in comparison to parental cells. On the other hand, radiosensitive 2A3 cells were arrested in G_2_/M-phase in response to 5 Gy irradiation, had a prominent accumulation of and slower resolution of DNA double-strand breaks, and no change in DNA damage signalling genes expression.

**Conclusions:**

We concluded that the emergence of the radioresistance in the established radioresistant isogenic cells can be at least partially attributed to the enhanced DNA double-strand break repair, altered expression of DNA damage signalling and repair genes. On the other hand, in radiosensitive isogenic cells the reduced ability to repair a high number of induced DNA double-strand breaks and no transcriptional response in DNA damage signalling genes indicate on a lack of adaptive response to irradiation. Altogether, our results confirmed that these isogenic cells with different radiosensitivity are an appropriate model to study the mechanisms of radioresistance.

## Background

Head and neck cancers are a heterogeneous group of tumours arising in multiple anatomic sub sites in the head and neck region, which are histologically mainly squamous cell carcinomas (SCCs) [1]. The major risk factor for the SCCs arising in the oral cavity, oropharynx, hypopharynx, and larynx is habitual exposure to tobacco and/or alcohol consumption, while human papillomavirus (HPV) infection is associated with oropharyngeal tumours [2]. Different etiology of head and neck SCC also reflects in different tumour biology. Namely, HPV-positive oropharyngeal tumours and cell lines are more sensitive to radiotherapy and chemotherapy treatment than HPV-negative [3–5]. Most commonly, a combination of surgery and (chemo) radiotherapy or upfront chemoradiotherapy is used for the treatment of non-metastatic head and neck SCC, as they result in a better effect than surgery or radiotherapy alone [6].

Despite the advances made in the management of head and neck SCC, a high locoregional recurrence or second primary cancer remain a serious problem in the previously treated area [7, 8]. Management of the recurrent or second primary SCC is often limited to palliative radiotherapy or systemic therapy, with a high risk of normal tissue toxicity, impaired quality of life and poor outcome [7].

The observed radioresistant tumour phenotype is a combination of numerous factors: disease clinical features, e.g. tumour stage and volume, microenvironment features, e.g. hypoxia, and intrinsic cellular features, e.g. enhanced DNA repair, modulation of cell cycle progression, free radical and reactive oxygen species (ROS) scavenging [9]. These intrinsic radioprotective mechanisms give a survival advantage to radioresistant cells, leading to treatment failure.

To improve therapeutic efficacy, it is crucial to understand and elucidate the underlying mechanisms of intrinsic cellular radioresistance. Isogenic cell lines with different radiosensitivity can be established from parental cell lines by repeated exposure to radiation and/or carcinogens [10]. Generally, these isogenic pairs include parental cells, and the established cells with a more radioresistant phenotype, while isogenic cells with a more radiosensitive phenotype are rarely commercially available [9, 11].

The aims of this study were to establish radioresistant isogenic cells from parental head and neck SCC cells, by repeated exposure to radiation, and to identify the differences in the irradiation response of the newly established radioresistant cells in comparison to isogenic parental and radiosensitive cells. Furthermore, the radio- and chemosensitivity, cell proliferation, cell cycle, induction and resolution of DNA double-strand breaks (DSBs) in parental, radioresistant and radiosensitive isogenic cells were evaluated. Additionally, the DNA damage signalling gene expression in these isogenic cells before and after irradiation, with a specific focus on the early response to irradiation, was elucidated.

## Methods

### Cell lines and cell culture conditions

Human pharyngeal SCC cell line FaDu (ATCC, HTB-43) was grown in Advanced Dulbecco’s Modified Eagle Medium (DMEM, Gibco, Thermo Fisher, MA, USA) supplemented with 5% fetal bovine serum (FBS, Gibco, Thermo Fisher), 10 mM L-glutamine (GlutaMAX, Gibco), penicillin (100 U/mL) (Grünenthal, Germany) and gentamicin (50 mg/mL) (Krka, Slovenia). Cells were routinely subcultured twice a week and incubated in a humidified atmosphere at 37 °C and 5% CO_2_.

The HPV-positive 2A3 cell line, a kind gift from Prof. Dadachova was established by transfection of FaDu cells with HPV16 oncogenes E6 and E7 [12]. 2A3 cells were grown in Advanced DMEM supplemented with 5% fetal bovine serum, 10 mM L-glutamine, penicillin (100 U/mL), gentamicin (50 mg/mL) and 1 mg/mL G418 disulfate salt solution (Sigma-Aldrich, MO, USA).

Authentication of FaDu, FaDu-RR, and 2A3 cells by short tandem repeats profiling was performed using CellCheck 16 – human (IDEXX BioAnalytics, Germany) for authentication of FaDu, and FaDu-RR cells (Additional file 1: Final report of laboratory examination) and CellCheck 9 – human (IDEXX BioAnalytics) for 2A3 cells (Additional file 2: Final report of laboratory examination). The genetic profile of the cell lines used for the study was identical to the publically available genetic profile of these cell lines.

### Establishment of isogenic radioresistant cells

**Fig. 1:**
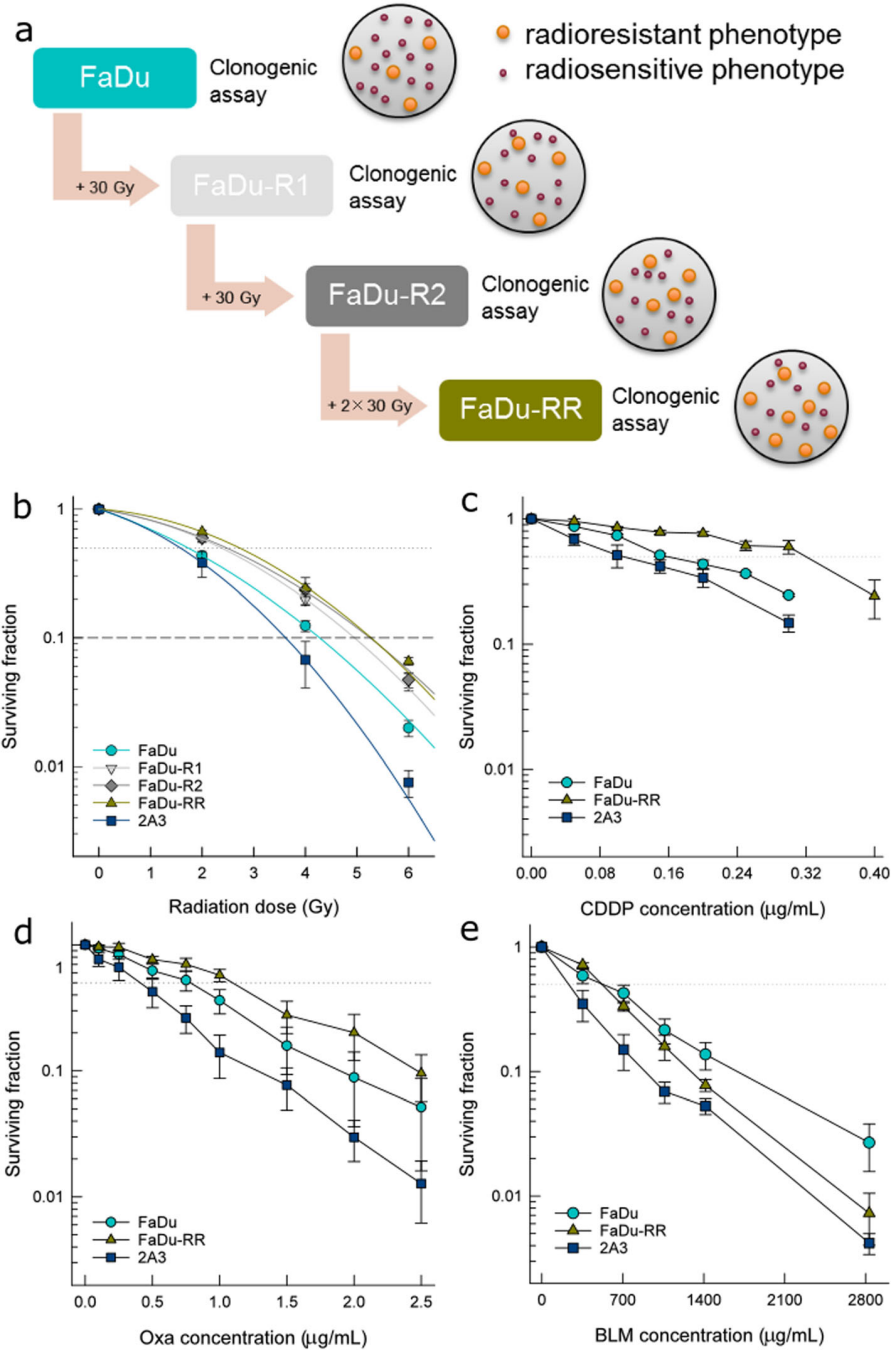
Radiosensitivity and chemosensitivity of isogenic head and neck squamous cell carcinoma cells with different radiosensitivity. **a** Schematic representation of the establishment of isogenic radioresistant FaDu-RR cells from parental FaDu cells by repeated exposure to one series (FaDu-R1), two series (FaDu-R2), and four series (FaDu-RR) of ionizing radiation; **b** Surviving fraction of isogenic parental FaDu, radioresistant FaDu-R1, FaDu-R2, and FaDu-RR, and radiosensitive 2A3 cells fitted to LQ model. Symbols are mean values (AM) ± standard error of the mean (SEM) from three independent experiments. The dashed line indicates the surviving fraction of 0.1 (D_10_ value). **c** Clonogenic survival of isogenic cells after exposure to (**c**) cisplatin (CDDP), **d** oxaliplatin (Oxa) and (**e**) bleomycin (BLM). The dotted line indicates a surviving fraction of 0.5. Symbols are AM ± SEM, *n* = 3

Radioresistant cells were derived from parental FaDu cells after repeated exposure to ionizing radiation using Gulmay MP1-CP225 X-ray unit (Gulmay Medical Ltd., UK) with a filter consisting of Cu thickness of 0.55 mm and Al thickness of 1.8 mm at the dose-rate 1.728 Gy/min. Cells were exposed to 2 Gy/day for 5 days/week for 3 weeks. After the first irradiation series, 6 different subclones were obtained, and their radiosensitivity was evaluated by clonogenic assay. The subclone FaDu-R1 with the highest surviving fraction at 2 Gy was selected for further irradiation. After additional irradiation, we were not able to select specific subclones, but whole cell population named FaDu-R2. Subline FaDu-R2 was further irradiated with two more series until radioresistant FaDu-RR cells were established (Fig. 1a). The recovered FaDu-R1, FaDu-R2, and FaDu-RR cells received a total dose of 30 Gy, 60 Gy, and 120 Gy, respectively. Since there is no consensus on how to consistently and accurately interpret an increase in cell survival of the irradiated cells as a sign of acquired radioresistant phenotype [9], we compared multiple parameters to describe the survival advantage of the recovered cells. Radiosensitivity was evaluated by comparison of the surviving fraction at 2 Gy (SF2), effective dose killing 50% of the cells (ED_50_), and the dose required to kill 90% of the cells (D_10_) values as well as α and β parameters. For comparison of ED_50_ and D_10_ values in radioresistant and parental cells, dose-modifying factor (DMF) was calculated as a ratio of dose in radioresistant cells and in the parental cells. Survival curves were fitted to a linear-quadratic (LQ) model $Sf={e}^{\left(-\alpha \times D-\beta \times {D}^2\right)}$ using Sigma Plot 13.0 (Systat Software Inc., CA, USA) to estimate α and β parameters. The established FaDu-RR cells were routinely evaluated for radioresistance and remained radioresistant for more than 4 years of normal maintenance (subculture, freezing and thawing). During this time, maintenance radiation (2 Gy/day for 5 days/week for 3 weeks) was applied to restore the radioresistant response in the established FaDu-RR cells when radioresistance decreased significantly. Before any experiments were performed, there was a recovery period of minimum 3 weeks to allow FaDu-RR to recover and continue normal proliferation.

### Radiosensitivity assay

Radiosensitivity of FaDu, 2A3 and FaDu-RR cells was evaluated by clonogenic assay. Cells were plated at appropriate concentrations, ranging from 400 cells for non-irradiated control up to 8000 cells for 10 Gy irradiation, in a 60-mm tissue culture dish and were irradiated at different doses ranging from 0 Gy to 10 Gy using Gulmay MP1-CP225 X-ray unit as described above. After 10 days, we fixed and stained with crystal violet/methanol solution and counted them. Surviving fraction for each cell line after exposure to ionizing radiation was calculated as a ratio of plating efficiency of treated cells and control cells. The experiment was repeated at least three times in triplicates. The ED_50_ was determined for each cell line and used to calculate the DMF.

### Chemosensitivity assay

The sensitivity of FaDu, 2A3 and FaDu-RR cells to cisplatin (CDDP), oxaliplatin (Oxa) and bleomycin (BLM) was evaluated by clonogenic assay. Crystalline CDDP powder (Platinol, Bristol Myers Squibb, Austria) was dissolved in sterile H_2_O. For each experiment, a fresh solution of final CDDP concentrations (0.05 μg/mL – 30 μg/mL), Oxa (Oxaliplatin Teva, Teva Pharma B. V., the Netherlands) concentrations (0.1 μg/mL - 2.5 μg/mL) and BLM (Bleomycin medac, Medac, Germany) concentrations (354 μg/mL - 2831 μg/mL) were prepared in DMEM. Cells were plated in the cell culture media with the drug for continuous drug exposure in a 60-mm tissue culture dish. After 10 days, the colonies were fixed, stained with crystal violet and counted. Plating efficiency and survival fraction were calculated as described in Radiosensitivity assay. The half-maximal inhibitory concentration (the concentration of a chemotherapeutic agent required for a 50% growth inhibition; IC_50_) was determined for each cell line and drug used. Each experiment was repeated 3 times in triplicates.

### Cell proliferation

Cells were plated on 96 well plates (500 per well) in Advanced DMEM with supplements as described above and allowed to attach for 3 h. Cell viability of FaDu, FaDu-RR, and 2A3 cells was determined by Presto Blue Cell Viability Reagent (Invitrogen, Thermo Fisher Scientific) according to the manufacturer’s recommendations every 24 h for 5 days. After 90-min incubation in a humidified atmosphere at 37 °C and 5% CO_2_, fluorescence was measured at excitation wavelength 535 nm and emission wavelength 595 nm with a microplate reader (Infinite 200, Tecan, Switzerland). Data were fitted to the exponential growth two-parameter equation *y* = *a* × *e*^*b* × *x*^ in SigmaPlot 13, where y is the measured fluorescence at time point t, a is the fluorescence at time point 0, b is a growth rate constant, and x is a time in hours. Doubling time (DT) for each cell line was then calculated from growth rate constant b as $=\frac{\ln 2}{b}$ .

### Cell cycle analysis

Flow cytometry was used to determine cell cycle distribution of isogenic FaDu, FaDu-RR and 2A3 cells after 5 Gy irradiation using Gulmay MP1-CP225 X-ray unit as described above. The samples were prepared following the standard procedure using fluorochrome DAPI (4′,6-diamidino-2-phenylindole-dihydrochloride), which stoichiometrically binds to the DNA *[*13*,* 14*]*. The samples were acquired using a flow cytometer Partec PAS II (Partec GmbH, Germany). During sample acquisition, at least 30,000 cells per sample were collected. DNA histograms of the number of cells against observed fluorescence intensity (Additional file 3: Figure S1) were obtained and analysed with MultiCycle AV DNA analysis software (Phoenics Flow Systems, Inc., CA, USA). Distributions of cells in G_1_, S and G_2_/M phases of the cell cycle were calculated. The experiment was repeated 3 times.

### Detection of apoptosis

Fold-induction of apoptosis was evaluated using the FITC Annexin V Apoptosis Detection Kit with 7-AAD (Biolegend, CA, USA) according to the manufacturer’s instructions, after no irradiation for control cells and after 5 Gy irradiation for irradiated cells using Gulmay MP1-CP225 X-ray unit as described above. After irradiation, cells were incubated for 5 h, 24 h, 48 h, and 72 h. Trypsinized cell suspension was combined with the collected cell media to include dead cells and centrifuged to remove the trypsin and cell media. Early apoptotic (Annexin V-positive and 7AAD-negative), late apoptotic and/or necrotic (Annexin V-positive and 7AAD-positive), necrotic (Annexin V-negative and 7AAD-positive) and viable (Annexin V-negative and 7AAD-negative) cells were analyzed by FACSCanto II flow cytometer (BD Biosciences, CA, USA) within 10 min (Additional file 4: Figure S2). Fold-induction of apoptosis was determined as the sum of early and late apoptotic (Annexin V-positive) cells at a specific time point, normalized to the sum of early and late apoptotic cells in control non-irradiated cells at 5 h. Fold-change of cell viability was determined as percentage of viable non-irradiated or 5 Gy irradiated cells at specific time point, normalized to the percentage of control non-irradiated viable cells at 5 h. The experiment was repeated 4 times.

### γH2AX immunofluorescence

FaDu, FaDu-RR, and 2A3 cells were seeded on glass coverslips (Knittelglass, Germany), incubated overnight and irradiated with 5 Gy using Gulmay MP1-CP225 X-ray unit as described above. Control non-irradiated cells (0 h) and irradiated cells at different time points after irradiation (15 min, 30 min, 45 min, 1 h, 2 h, 5 h, 24 h, 48 h and 72 h), were fixed and stained for γH2AX immunofluorescence as described previously *[*15*]*. γH2AX foci were viewed under the Olympus BX51 fluorescence microscope at 100-fold magnification. Images were analysed using Image J 1.51 g software tool (NIH, MD, USA) to score γH2AX foci/nuclei. First, DAPI-stained nuclei were selected to omit nuclear debris, apoptotic-like nuclei, and enlarged nuclei. Then, nuclei outlines were overlaid on γH2AX foci image to count the number of foci in each nucleus. Foci were counted semi-automatically by empirically setting a threshold level for γH2AX foci. For each condition, at least 250 nuclei were analysed and data from three independent experiments were pooled together. A cut-off value for γH2AX-positive cells was 90th percentile of background γH2AX foci in control non-irradiated cells. To evaluate the kinetics of γH2AX foci resolution, the experimental data were fitted to two-phase exponential decay equation $y= Plateau+ SpanFast\times {e}^{K_{fast}\times x}+ SpanSlow\times {e}^{K_{slow}\times x}$ to evaluate the half-life of γH2AX foci in the fast and slow phases of DSB repair *[*16*]*.

### Gene expression analysis

To study the expression of genes involved in DNA damage signalling, Human DNA Damage Signalling Pathway RT^2^ ProfilerTM PCR Array (PAHS-029Z, Qiagen, Germany) was used. A list of 84 pathway specific and 5 house-keeping genes on this array is available in Additional file 5: Table S1. Genomic DNA control, reverse transcription control, and positive PCR controls were also included on the array. Cells were irradiated using a Gulmay MP1-CP225 X-ray unit as described above. Total RNA was isolated from non-irradiated and 5 Gy-irradiated FaDu, 2A3 and FaDu-RR cells 5 h after irradiation, by using RNeasy Plus Mini Kit (Qiagen). Briefly, RNA concentration and sample purity (A_260/280_) were determined spectrophotometrically and 2 μg total RNA was used to synthesize cDNA using RT^2^ First Strand Kit (Qiagen). Real time reverse transcription polymerase chain reaction (RT-PCR) was carried out on QuantStudio 3 Real-time PCR System (Applied Biosystems, USA) using RT^2^ qPCR Sybr Green ROX Mastermix (Qiagen). RT-PCR cycling conditions were 10 min at 95 °C for activation of HotStart DNA Taq Polymerase, followed by 40 amplification cycles of 15 s at 95 °C and 2 min at 60 °C. RT-PCR specificity was verified by melting curve analysis, immediately after each RT-PCR run. Melt curve program consisted of 1 min at 95 °C, 2 min at 65 °C and dissociation step from 65 °C to 95 °C at 2 °C/minute.

Data were analyzed on the GeneGlobe Data Analysis Center (Qiagen). Data were normalized to automatically selected housekeeping genes with the most stable expression. For comparison of non-irradiated control cells, data were normalized to the gene expression of housekeeping genes *ACTB*, *B2M* and *RPLP0*, and for comparison of 5 Gy-irradiated cells, data were normalized to the gene expression of housekeeping genes *ACTB* and *RPLP0*. Fold change in gene expression was calculated using the ΔΔCT method [8]. We used 1.5 fold-change in gene expression as a threshold and *p*-values less than 0.05 to identify significantly different gene expression. For statistical analysis, Student’s t-test (two-tail distribution and equal variances between the two samples) was used on the replicate 2^–ΔΔCT^ values for each gene in each treatment group compared to the control group from 3 independent experiments.

Additionally, we used GeneMANIA and Reactome Pathway Database for to help predict the functions of the identified differentially expressed genes in isogenic cells with different radiosensitivity and in response to 5 Gy irradiation in these isogenic cells. GeneMANIA was used to identify functions of differentially expressed genes and visualize interactions between the differentially expressed genes [17]. These interactions included physical interaction (two gene products are linked if they were found to interact in a protein-protein interaction study), predicted interactions (two proteins are predicted to interact if their orthologs are known to interact in another organism), co-expression (two genes are linked if their expression levels are similar across conditions in a gene expression study), genetic interaction (two genes are functionally associated if the effects of perturbing one gene were found to be modified by perturbations to a second gene), pathway interactions (two gene products are linked if they participate in the same reaction within a pathway), co-localization (two genes are linked if they are both expressed in the same tissue or if their gene products are both identified in the same cellular location), and shared protein domains (two gene products are linked if they have the same protein domain). In addition, Reactome Pathway Database was used for the visualization and comparison of the signalling pathways affected by the differential gene expression in isogenic cells with different radiosensitivity and by exposure to 5 Gy irradiation [18].

### Statistics

SigmaPlot 13.0 (Systat Software Inc., San Jose, CA, USA) was used for statistical analysis. Data were tested for normality of distribution using the Shapiro-Wilk test. For normally distributed data, arithmetic mean (AM) and standard error of the mean (SEM) were calculated. Unless otherwise stated, all multiple comparisons were tested using the Holm-Sidak method after One Way ANOVA. Differences were considered significant for *P* values less than 0.05.

## Results

### Radiosensitivity of isogenic cells

After repeated exposure to radiation, different isogenic radioresistant cells (Fig. 1a), derived from human pharyngeal SCC FaDu, were established. Their radiosensitivity (Fig. 1b) was evaluated by calculating SF2, ED_50_, D_10_, α, and β parameters and α/β ratio of the LQ model (Additional file 6: Table S2). FaDu-RR cells had a significantly increased SF2, ED_50_, and D_10_ values, and significantly decreased α and α/β parameters in comparison to parental FaDu cells and isogenic 2A3 cells. The latter had significantly decreased ED_50_ and increased α and α/β parameters compared to parental FaDu and isogenic FaDu-RR cells. Based on these results, FaDu-RR cells displayed a radioresistant, and 2A3 cells a radiosensitive phenotype in comparison to parental FaDu cells. Plating efficiency of control non-irradiated FaDu, FaDu-RR, and 2A3 cells was 46.1, 46.9, and 45.6% respectively, and did not differ significantly between the cell lines.

### Chemosensitivity

In addition to radioresistance, FaDu-RR cells displayed cross-resistance to CDDP (Fig. 1c) and Oxa (Fig. 1d), but not to BLM (Fig. 1e). Namely, IC_50_ values for CDDP and Oxa in radioresistant FaDu-RR cells were potentiated 1.8-fold and 1.5-fold, respectively, while no difference in BLM sensitivity was observed, when compared to the parental cell line (Table 1). In comparison to the latter, increased sensitivity to CDDP, Oxa and BLM was also indicated in the radiosensitive 2A3 cells.

**Table 1:** Half-maximal inhibitory concentration (IC_50_) values of isogenic cells to chemotherapeutics

Cytotoxic drug (μg/mL)	FaDu	FaDu-RR	Fold-potentiation^a^	2A3	Fold-potentiation^a^
Cisplatin	0.16 ± 0.007	0.284 ± 0.02^b^	1.8	0.1 ± 0.03	0.7
Oxaliplatin	0.75 ± 0.15	1.12 ± 0.15^c^	1.5	0.41 ± 0.13	0.5
Bleomycin	551 ± 132	521 ± 18	0.9	249 ± 63	0.5

^a^Fold-potentiation compared to parental FaDu cell line. ^b^ significant difference compared to FaDu and 2A3 cells. ^c^ significant difference compared to 2A3. Values are AM ± SEM

### Cell proliferation

**Fig. 2:**
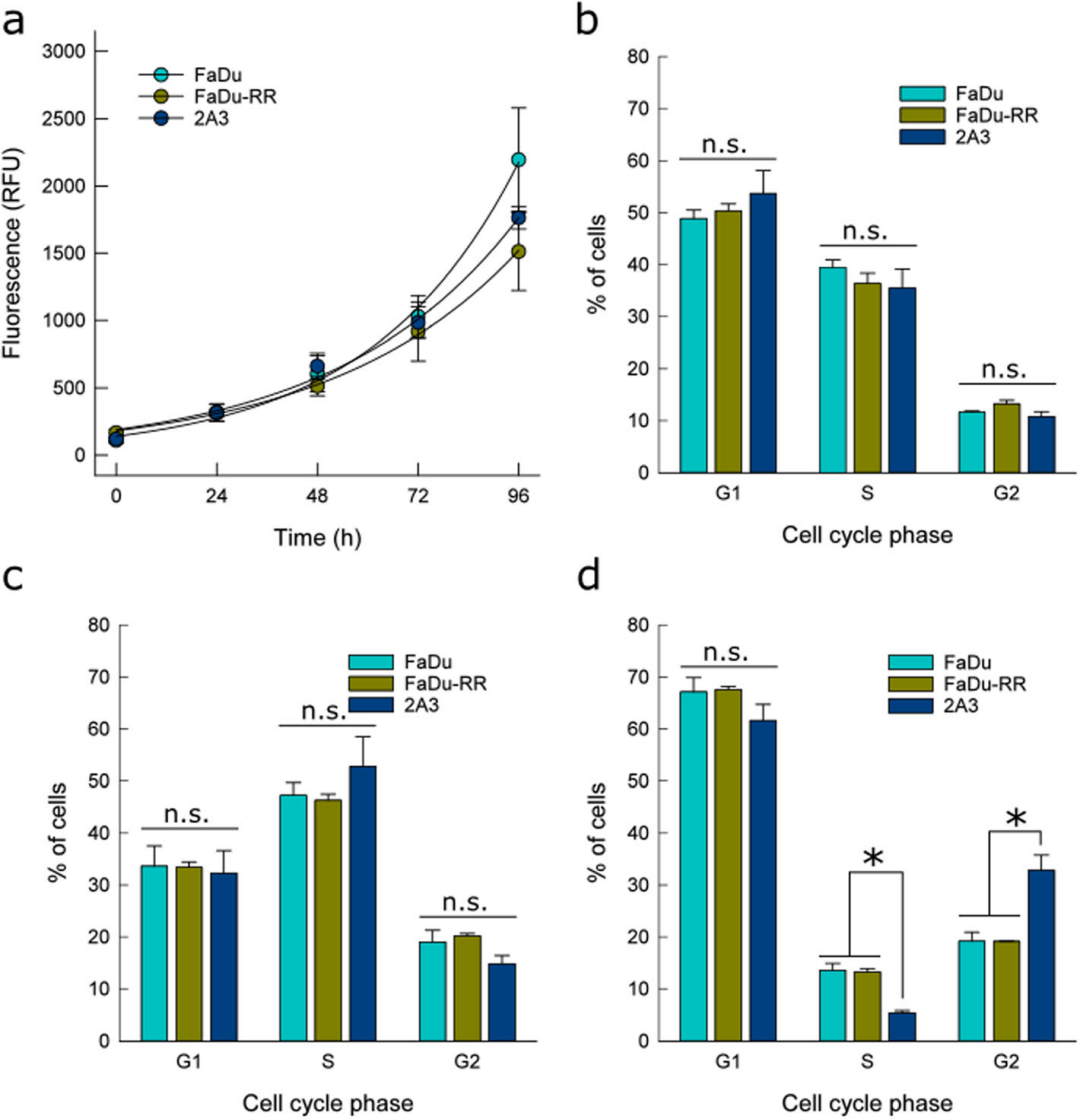
Cell proliferation and cell cycle distribution of isogenic head and neck squamous cell carcinoma cells. **a** No difference in cell proliferation of isogenic cells with different radiosensitivity was observed. **b** Cell cycle phase distribution before irradiation, **c** 5 h after 5 Gy irradiation, and **d** 24 h after 5 Gy irradiation. Symbols and bars AM ± SEM from three independent experiments. * indicates significant difference; n.s. – non-significant

Similar, statistically non-significant growth rates and DTs of isogenic FaDu, FaDu-RR and 2A3 cells (Fig. 2a) were observed. DT of parental FaDu cells was 24.5 ± 2.3 h, of radioresistant FaDu-RR cells 33.8 ± 1.5 h, and of radiosensitive 2A3 cells 29.6 ± 2.4 h.

### Cell cycle distribution

Before irradiation, the cell cycle distribution of isogenic FaDu, FaDu-RR, and 2A3 cells did not differ significantly between cells (Fig. 2b). Primarily, cells were in G_1_ phase (51%), followed by those in the S phase (37%) and G_2_/M phase (12%). We did not observe difference in chromosome number in these isogenic cell lines (Additional *file* 3*:* Figure *S*1). Five hours post-irradiation, cell cycle distribution among isogenic cells was preserved (Fig. 2c), although different from the distribution of non-irradiated cells. The majority of cells were in S phase, while the percentage of cells in G_1_ phase decreased and increased in G_2_/M phase (Additional file 7: Figure S3). However, 24 h after 5 Gy irradiation, the percentage of G_1_ cells was similar or higher as before irradiation. Specifically for FaDu and FaDu-RR cells the difference in G_1_ cells was significantly higher after 24 h, while in 2A3 cells, there were more G_1_ cells after 24 h, however the increase was not significant. Meanwhile, the minority of cells were in S phase. Among isogenic cell lines, statistically significant differences in cell cycle distribution were observed. In comparison to the parental FaDu and radioresistant FaDu-RR cells, the percentage of cells in S phase was the lowest in radiosensitive 2A3 cells (5%), while in the G_2_/M-phase it was the highest (33%) (Fig. 2d).

### Radiation-induced apoptosis

**Fig. 3:**
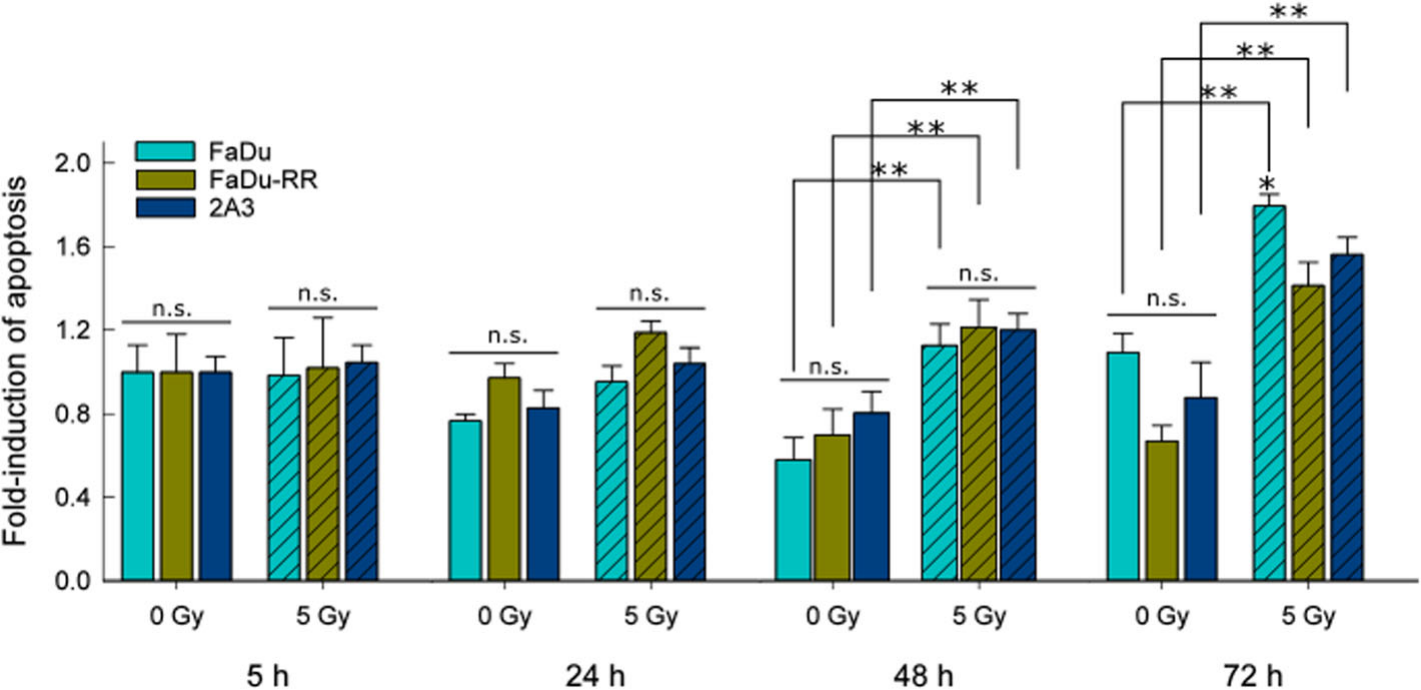
Fold-induction of apoptosis after 5 Gy irradiation in isogenic FaDu, FaDu-RR, and 2A3 cells. Significantly higher fold-induction of apoptosis was observed in FaDu 72 h after irradiation, in comparison to FaDu-RR or 2A3 cells. Bars are AM ± SEM from three independent experiments. ** indicates a significant difference between 5 Gy irradiated and control non-irradiated cells; * indicates a significant difference compared to FaDu-RR and 2A3 cells; n.s. – non-significant

We observed very low levels of early and late apoptotic cells (Additional file 8: Table S3). To identify differences in response to irradiation, fold-induction of apoptosis was determined for each specific time point relative to control non-irradiated cells at 5 h. In non-irradiated control cells, no induction of apoptosis was detected, while in isogenic FaDu, FaDu-RR and 2A3 cells (Fig. 3), apoptosis was induced by irradiation. In general, significantly higher fold-induction of apoptosis was observed 72 h after irradiation in parental FaDu and radiosensitive 2A3 cells, compared to the earlier time points (5, 24 and 48 h), while fold-induction of apoptosis in radioresistant FaDu-RR was sustained in time (Additional file 9: Figure S4).

These results parallel the cell viability of isogenic cell lines after exposure to 5 Gy irradiation. While FaDu and 2A3 fold-change of cell viability was significantly reduced 72 h after 5 Gy irradiation compared to non-irradiated cells at all timepoints, FaDu-RR fold-change of cell viability was less affected, and was significantly lower only in comparison to non-irradiated FaDu-RR cells 72 h after plating (Additional file 10: Figure S5).

### γH2AX immunofluorescence

**Fig. 4:**
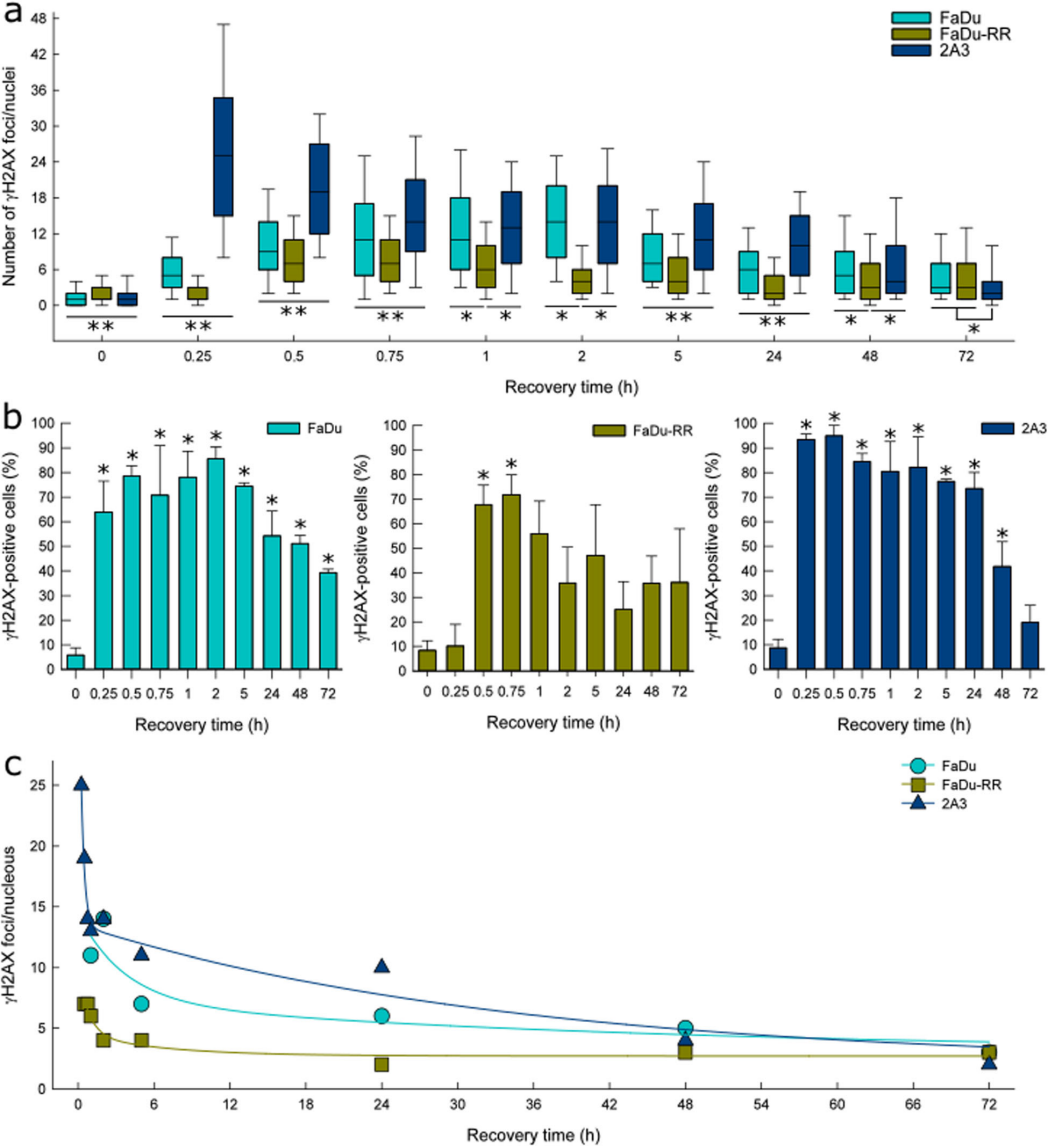
γH2AX foci in isogenic FaDu, FaDu-RR and 2A3 cells after 5 Gy irradiation. **a** A median number of γH2AX foci in isogenic cells determined by γH2AX immunofluorescence at various recovery times after irradiation. Box plots are median values with 25th and 75th percentile with 10th and 90th percentiles as bottom and top whiskers. ** indicates a significant difference in γH2AX foci number between isogenic FaDu, FaDu-RR, and 2A3 cells; * indicates a significant difference in γH2AX foci number. **b** The fraction of γH2AX-positive isogenic cells at various recovery times after irradiation. Bars are AM ± SEM from three independent experiments. * indicates a significant difference in the fraction of in γH2AX-positive cells compared to control non-irradiated cells. **c** Kinetics of γH2AX foci resolution in isogenic cells. Experimental data were fitted to a two-phase exponential decay equation. Symbols are the median values of pooled data from three independent experiments

A different temporal expression of γH2AX foci in isogenic cells (Fig. 4a) was observed. Induction of foci was detected as early as 15 min after irradiation in the parental FaDu and radiosensitive 2A3 cells, whereas, in radioresistant FaDu-RR cells, significant induction was observed 30 min after irradiation. Accumulation of γH2AX foci was most prominent in radiosensitive 2A3 cells, in which the peak was observed already 15 min after irradiation. After 30 to 45 min, the peak of γH2AX foci was observed also in radioresistant FaDu-RR cells, while in FaDu cells, the γH2AX foci accumulated more slowly and reached a peak expression 2 h after irradiation. The median number of foci observed at the peak also differed between the cells, ranging from 25 in radiosensitive 2A3 cells, 14 in parental FaDu cells to 7 foci in radioresistant FaDu-RR cells. The number of residual γH2AX foci 72 h after irradiation remained significantly higher compared to control non-irradiated isogenic cells.

Furthermore, differences were also in the percent of γH2AX-positive cells (Fig. 4b) and in the γH2AX foci resolution (Fig. 4c). A maximum of γH2AX-positive cells was observed at the peak of γH2AX formation. Namely, 86 ± 5% of parental FaDu cells, 72 ± 8% of radioresistant FaDu-RR cells, and 93 ± 2% of radiosensitive 2A3 cells were γH2AX-positive at the peak of γH2AX foci. In parental FaDu and radiosensitive 2A3 cells, a fraction of γH2AX-positive cells remained significantly elevated up to 48 h after irradiation, while in radioresistant FaDu-RR cells a fraction of γH2AX-positive cells was significantly increased only between 30 and 45 min after irradiation. In the fast phase of DNA DSB repair, radioresistant FaDu-RR and radiosensitive 2A3 cells had a short half-life of γH2AX foci (34 and 11 min, respectively), while the half-life of γH2AX foci in parental FaDu cells was 2.5 h. In the slow phase of DNA DSB repair, radioresistant FaDu-RR cells also had the shortest half-time of γH2AX foci (4.5 h), while parental FaDu and radiosensitive 2A3 had much longer half-time of γH2AX foci (31.8 and 24.1 h, respectively).

Additionally, significantly enlarged cell nuclei, with more than 2-fold increase in the nuclear area, 48 and 72 h after irradiation (Additional file 11: Figure S6) were observed. The fraction of cells with significantly enlarged nuclei was the highest in radiosensitive 2A3 cells (40% at 48 h and 46% at 72 h), intermediate in parental FaDu cells (29.2% at 48 h and 31.5% at 72 h) and the lowest in radioresistant FaDu-RR cells (22.3% at 48 h and 12.3% at 72 h). These cells also had a persistently high number of residual γH2AX foci (21 and 15 γH2AX foci in FaDu, 13 and 17 γH2AX foci in FaDu-RR, and 17 and 16 γH2AX foci in 2A3 at 48 and 72 h, respectively).

### DNA damage signalling and repair (DSR) gene expression

The expression of 84 relevant genes involved in DNA DSR was determined in parental FaDu, radioresistant FaDu-RR, and radiosensitive 2A3 cells, before and 5 h after 5 Gy irradiation.

**Fig. 5:**
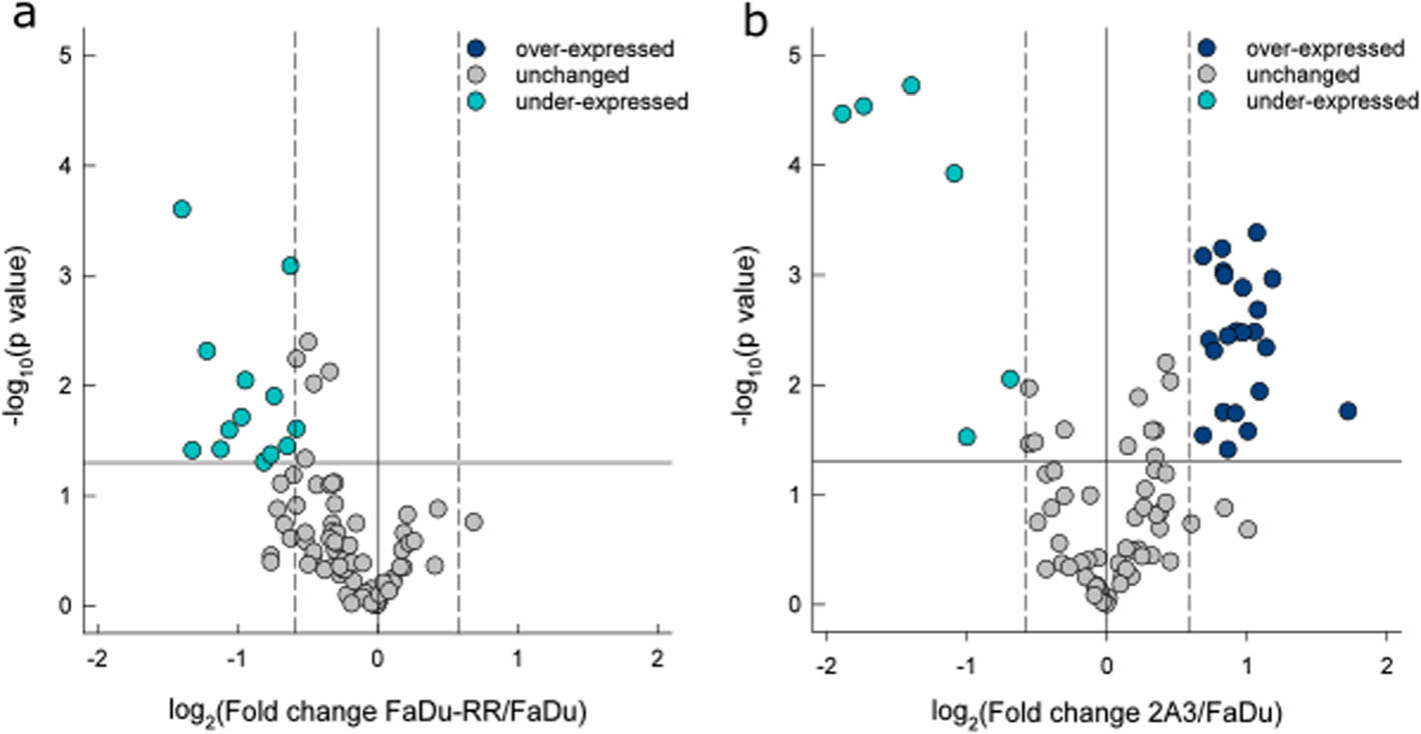
DNA damage signalling and repair gene expression in non-irradiated isogenic FaDu, FaDu-RR, and 2A3 cells. **a** Gene expression in non-irradiated radioresistant FaDu-RR relative to non-irradiated parental FaDu cells; **b** gene expression in non-irradiated radiosensitive 2A3 cells relative to parental FaDu cells. Volcano plots show the fold change in gene expression and statistical significance (*p*-value). The horizontal line shows the statistical significance threshold (p-value < 0.05). Two vertical dashed lines show the threshold of over-expressed (right) and under-expressed genes (left), while the solid vertical line shows no change in gene expression. Symbols represent the mean gene expression of each tested gene in FaDu-RR and 2A3 cells relative to parental FaDu cells from three independent experiments

An altered DNA DSR gene expression between non-irradiated parental FaDu, radioresistant FaDu-RR, and radiosensitive 2A3 cells was observed. Namely, of the 84 DNA DSR genes included in the analysis, 12 genes were under-expressed in radioresistant FaDu-RR cells (Fig. 5a). In radiosensitive 2A3 cells, 22 genes were over-expressed and 6 genes were under-expressed for a total of 28 differentially expressed genes (Fig. 5b). Of these differentially expressed genes, two genes, *BLM* and *RNF168*, were both under-expressed in FaDu-RR and 2A3 cells, while no genes were over-expressed in both FaDu-RR and 2A3 cells. We identified four genes (*FANCD2, GADD45A, H2AFX,* and *XRCC2*) which were under-expressed in radioresistant FaDu-RR cells, yet over-expressed in radiosensitive 2A3 cells. Summary of the DNA DSR gene expression in non-irradiated FaDu-RR and 2A3 cells is provided in Table 2.

**Table 2:** Basal gene expression of genes involved in DNA damage signaling pathway in non-irradiated isogenic radioresistant FaDu-RR and radiosensitive 2A3 cells compared to non-irradiated parental FaDu cells

Gene^a^	FaDu-RR^b^	P value^c^	2A3^d^	P value^c^	DNA damage signaling pathways
*APEX1*			2.11	0.002	BER
*ATM*			2.08	0.003	ATM/ATR signaling, DSB repair, apoptosis, cell cycle
*BARD1*			1.66	0.004	ATM/ATR signaling, other DNA repair genes, apoptosis
*BBC3*			−1.99	0.03	apoptosis
*BLM*	− 2.60	0.0003	−3.35	0.00003	DSB repair
*CHEK1*			1.96	0.001	ATM/ATR signaling, DSB repair, cell cycle
*CIB1*			−3.69	0.00003	other DNA repair genes, apoptosis
*DDB2*			1.78	0.02	NER
*ERCC2*	−2.35	0.005			NER
*FANCD2*	−1.74	0.05	1.78	0.0010	ATM/ATR signaling, other DNA repair genes
*FEN1*			1.79	0.001	BER
*GADD45A*	−2.19	0.04	3.30	0.02	other DNA repair genes
*H2AFX*	−1.92	0.009	1.61	0.03	ATM/ATR signaling, DSB repair
*HUS1*	−2.10	0.03			ATM/ATR signaling, DSB repair
*MBD4*	−1.54	0.0008			BER
*MLH1*			2.13	0.01	DSB repair, mismatch repair
*MSH3*			1.77	0.0006	mismatch repair
*OGG1*			1.70	0.005	NER, BER
*PCNA*	−1.57	0.04			NER, BER, mismatch repair
*PMS1*			1.90	0.003	mismatch repair
*PNKP*			−1.60	0.009	NER
*PPM1D*			1.82	0.04	cell cycle
*PRKDC*			1.89	0.02	DSB repair, apoptosis
*RAD1*			2.10	0.0004	ATM/ATR signaling, other DNA repair genes
*RAD17*			1.96	0.003	ATM/ATR signaling, other DNA repair genes
*RAD18*			1.82	0.004	other DNA repair genes
*RBBP8*			2.27	0.001	ATM/ATR signaling, other DNA repair genes
*REV1*			1.61	0.0007	other DNA repair genes
*RNF168*	−1.69	0.04	−2.13	0.0001	ATM/ATR signaling, other DNA repair genes
*SUMO1*			−2.60	0.00002	other DNA repair genes
*TOPBP1*	−1.68	0.01			ATM/ATR signaling, other DNA repair genes
*UNG*			2.20	0.005	BER
*XRCC2*	−2.47	0.04	2.01	0.03	DSB repair
*XRCC3*	−1.96	0.02			other DNA repair genes

^a^ Gene symbol according to HUGO Gene Nomenclature Committee; ^b^ Fold regulation in gene expression in radioresistant FaDu cells in comparison to parental FaDu cells. Positive fold regulation values indicate gene over-expression, negative fold regulation values indicate gene under-expression; ^c^ The P values are calculated based on a Student’s t-test of the replicate 2^(− ΔΔ CT)^ values for each gene in the parental FaDu cells and radioresistant FaDu-RR or radiosensitive 2A3 cells, and differences in gene expression were considered significant for *P* values less than 0.05; ^d^ Fold regulation in gene expression in radiosensitive 2A3 cells in comparison to parental FaDu cells. *BER* base excision repair, *DSB* double-strand break, *NER* nucleotide excision repair

Functional analysis associated the 12 under-expressed genes in radioresistant FaDu-RR with response to ionizing radiation, response to radiation, cellular response to ionizing radiation, DNA recombination, regulation of DNA metabolic process, positive regulation of DNA metabolic process, and DNA N-glycosylase activity (Additional file 12: Figure S7a). Interactions between these differentially expressed genes in radioresistant FaDu-RR cells included physical and predicted interactions, co-expression, pathways, co-localization and shared protein domains (Additional file 12: Figure S7b).

Differentially expressed genes in radiosensitive 2A3 cells were associated with response to radiation, DNA recombination, DSB repair, damaged DNA binding, response to ionizing radiation, DNA damage checkpoint and DNA integrity checkpoint (Additional file 12: Figure S7c). These differentially expressed genes in radiosensitive 2A3 cells are connected through physical and predicted interactions, co-expression, genetic interactions, pathways, co-localization and shared protein domains (Additional file 12: Figure S7d).

We visualized the signalling pathways in which the differentially expressed genes were involved with Reactome Pathway Database. Differentially expressed genes in radioresistant FaDu-RR and radiosensitive 2A3 cells were involved in DNA DSB repair, base excision repair, nucleotide excision repair (NER), mismatch repair, and Fanconi anemia pathway, as well as cell cycle checkpoints, meiosis, chromosome maintenance and mitotic G_1_-G_1_/S phases (Additional file 12: Figure S7e). Specifically in radiosensitive 2A3 cells, one under-expressed genes was involved in the intrinsic pathway of apoptosis.

**Fig. 6:**
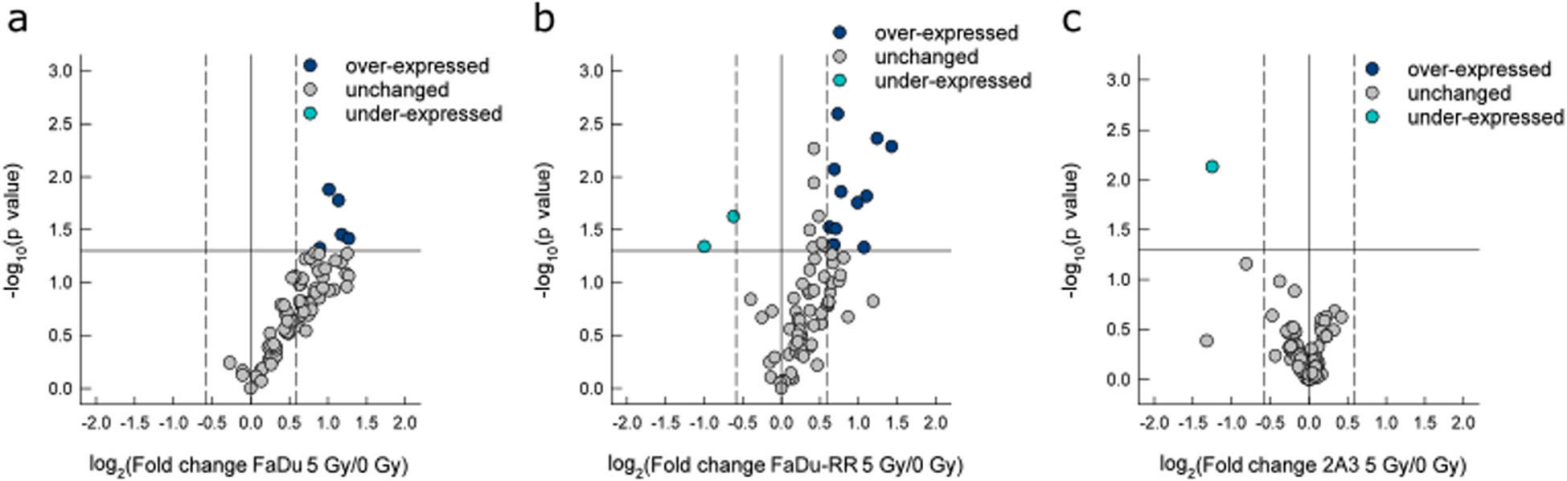
DNA damage signalling and repair gene expression in irradiated isogenic FaDu, FaDu-RR, and 2A3 cells. **a** Gene expression in 5 Gy-irradiated parental FaDu cells relative to non-irradiated FaDu cells; **b** gene expression in 5 Gy-irradiated radioresistant FaDu-RR relative to non-irradiated FaDu-RR cells; **c** gene expression in 5 Gy-irradiated radioresistant 2A3 cells relative to non-irradiated 2A3 cells. Volcano plots show the fold change in gene expression and statistical significance (p-value). The horizontal line shows the statistical significance threshold (p-value < 0.05). Two vertical dashed lines show the threshold of over-expressed (right) and under-expressed genes (left). The solid vertical line shows no change in gene expression. Symbols represent the mean gene expression of each tested gene in 5 Gy-irradiated cells relative to pertinent non-irradiated cells from three independent experiments

Differences were observed also in radiation-induced gene expression of DNA DSR genes 5 h after irradiation in isogenic FaDu, FaDu-RR and 2A3 relative to non-irradiated control cells. Specifically, 5 genes were over-expressed in parental FaDu cells (Fig. 6a), whereas in radioresistant FaDu-RR cells, 12 genes were over-expressed, and 2 genes were under-expressed for a total of 14 differentially expressed genes (Fig. 6b). In radiosensitive 2A3 cells, 1 gene was under-expressed (Fig. 6c). Of these differentially expressed genes, 4 genes (*BLM*, *GADD45A*, *MDC1*, *PMS1*) were over-expressed in both parental FaDu and radioresistant FaDu-RR cells. Specifically, in radioresistant FaDu-RR cells, 8 genes (*ATR*, *XPA*, *MSH3*, *MCPH1*, *H2AFX*, *NBN*, *ERCC2*, *XRCC2*) were over-expressed and 2 genes (*BBC3*, *CRY1*) were under-expressed. One gene, *XRCC3*, was under-expressed specifically in radiosensitive 2A3 cells after 5 Gy irradiation. A summary of the radiation-induced DNA DSR gene expression in FaDu, FaDu-RR, and 2A3 cells is provided in Table 3.

**Table 3:** Radiation-induced gene expression of genes involved in DNA damage signaling pathway in isogenic parental FaDu, radioresistant FaDu-RR and radiosensitive 2A3 cells 5 h after 5 Gy irradiation compared to non-irradiated parental FaDu, radioresistant FaDu-RR and radiosensitive 2A3 cells, respectivelly

Gene^a^	FaDu^b^	P value^c^	FaDu-RR^b^	P value^c^	2A3^b^	P value^c^	DNA damage signaling pathways
*ATR*			1.71	0.01			ATM/ATR signaling, DSB repair, apoptosis, cell cycle
*BBC3*			−2.00	0.05			apoptosis
*BLM*	2.26	0.04	2.69	0.005			DSB repair
*CRY1*			−1.54	0.02			other DNA repair genes
*ERCC2*			1.54	0.03			NER
*GADD45A*	2.02	0.01	2.36	0.004			other DNA repair genes
*H2AFX*			1.63	0.03			ATM/ATR signaling, DSB repair
*MCPH1*			1.66	0.003			cell cycle
*MDC1*	1.86	0.05	1.59	0.04			DSB repair, cell cycle
*MSH3*			1.60	0.04			mismatch repair
*NBN*			1.61	0.009			DSB repair
*PMS1*	2.20	0.02	2.15	0.02			mismatch repair
*PRKDC*	2.41	0.04					DSB repair, apoptosis
*XPA*			1.99	0.02			NER
*XRCC2*			2.10	0.05			DSB repair
*XRCC3*					−2.40	0.007	other DNA repair genes

^a^ Gene symbol according to HUGO Gene Nomenclature Committee; ^b^ Fold regulation in gene expression in 5 Gy-irradiated cells in comparison to non-irradiated cells. Positive fold regulation values indicate gene over-expression, negative fold regulation values indicate gene under-expression; ^c^ The P values were calculated based on a Student’s t-test of the replicate 2^(− ΔΔ CT)^ values for each gene in the 5 Gy-irradiated and non-irradiated cells, and differences in gene expression were considered significant for P values less than 0.05. *DSB* double-strand break, *NER* nucleotide excision repair

Functional analysis of the differentially expressed genes in 5 Gy-irradiated parental FaDu cells associated these genes with DSB repair, DNA recombination, cellular response to ionizing radiation, recombinational repair, single-stranded DNA binding, DSB repair via homologous recombination and regulation of cyclin-dependent protein serine/threonine kinase activity (Additional file 13: Figure S8a). We identified physical and predicted interactions, co-expression, and co-localization of differentially expressed genes in irradiated parental FaDu cells (Additional file 13: Figure S8b). In irradiated radioresistant FaDu-RR cells, differentially expressed genes were associated with DNA recombination, DNA integrity checkpoint, DNA damage checkpoint, response to radiation, recombinational repair, DNA-dependent ATPase activity and DSB repair via homologous recombination (Additional file 13: Figure S8c) and were associated through physical and predicted interactions, co-expression and co-localization, genetic interaction, pathways and shared protein domains (Additional file 13: Figure S8d). Over-represented pathways in irradiated parental FaDu and radioresistant FaDu-RR cells were identified with Reactome Pathway Database, indicating differences in DNA repair, cell cycle and programmed cell death (Additional file 13: Figure S8e). The differences in DNA repair included DNA DSB repair, mismatch repair, and NER. Regarding DNA DSB repair, radioresistant FaDu-RR cells had more over-expressed genes involved in non-homologous end joining, homology-directed repair, and DNA DSB response than parental FaDu cells. Similarly, radioresistant FaDu-RR cells had more over-expressed genes involved in the cell cycle than parental FaDu cells. Specifically, in radioresistant FaDu-RR cells, *BBC3* gene, involved in the intrinsic pathway of apoptosis, was under-expressed. On the other hand, radiosensitive 2A3 cells had under-expressed *XRCC3* gene involved in homology-directed repair, whereas no change in expression of genes involved in cell cycle and programmed cell death was observed.

**Fig. 7:**
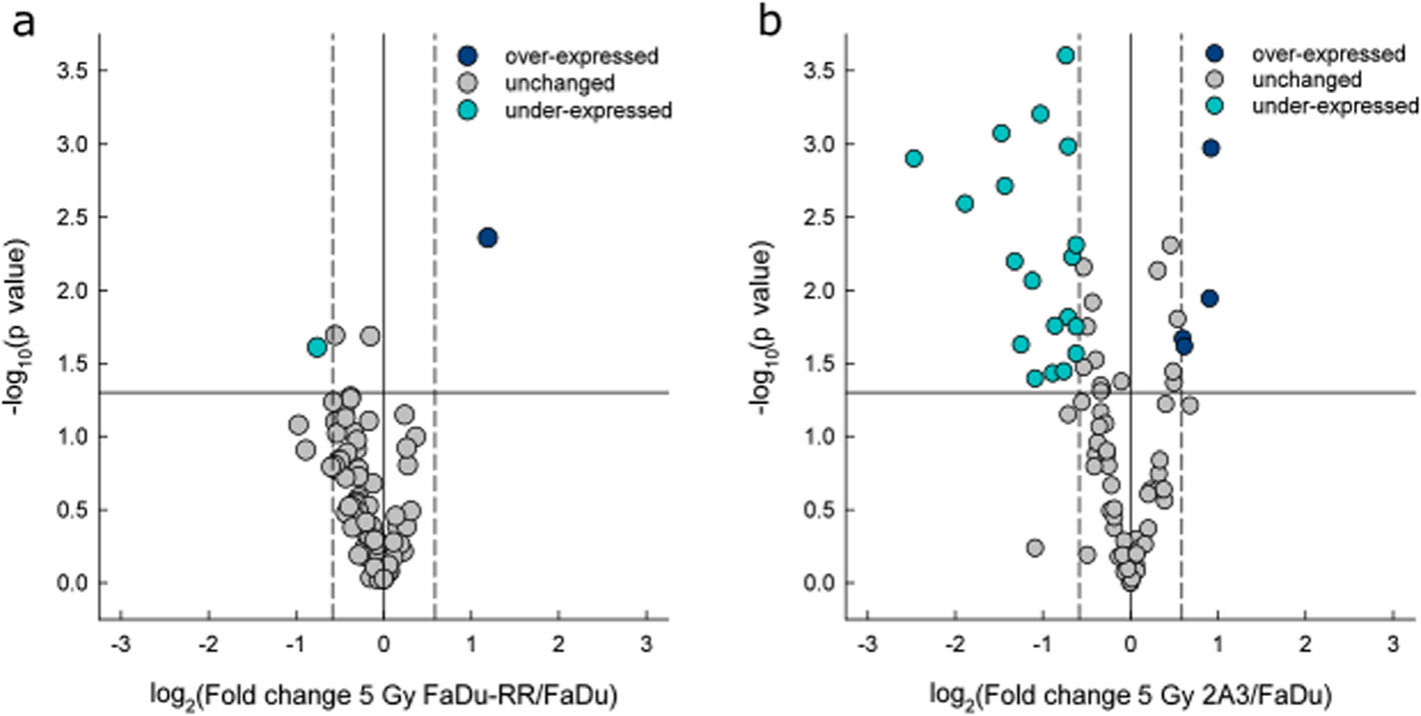
DNA damage signalling and repair gene expression in irradiated isogenic FaDu, FaDu-RR, and 2A3 cells. **a** Gene expression in 5 Gy-irradiated FaDu-RR cells relative to 5 Gy-irradiated FaDu cells. **b** Gene expression in 5 Gy-irradiated 2A3 cells relative to 5 Gy-irradiated FaDu cells. Volcano plots show the fold change in gene expression and statistical significance (p-value). The horizontal line shows the statistical significance threshold (p-value < 0.05). Two vertical dashed lines show the threshold of over-expressed (right) and under-expressed genes (left). The solid vertical line shows no change in gene expression. Symbols represent the mean gene expression of each tested gene in 5 Gy-irradiated radioresistant FaDu-RR and radiosensitive 2A3 cells relative to 5 Gy-irradiated parental FaDu cells from three independent experiments

By direct comparison of gene expression in 5 Gy-irradiated radioresistant FaDu-RR cells and parental FaDu cells, 2 differentially expressed genes in FaDu-RR cells were detected, of which *BLM* gene was under-expressed and *XPA* gene was over-expressed (Fig. 7a). On the other hand, 5 Gy irradiated radiosensitive 2A3 cells had 25 differentially expressed genes in comparison to 5 Gy irradiated parental FaDu cells, of which 4 genes were over-expressed and 21 genes were under-expressed (Fig. 7b). Of these differentially expressed genes, the *BLM* gene was under-expressed in both radioresistant FaDu-RR and radiosensitive 2A3 cells, while the *XPA* gene was over-expressed in radioresistant FaDu-RR, but under-expressed in radiosensitive 2A3 cells. Summary of the DNA DSR gene expression in 5 Gy-irradiated FaDu-RR and 2A3 cells relative to 5 Gy-irradiated FaDu cells is provided in Table 4.

**Table 4:** DNA damage signaling gene expression in 5 Gy-irradited radioresistant FaDu-RR cells and 5 Gy-irradiated radiosenstive 2A3 cells compared to 5 Gy-irradiated parental FaDu cells

Gene^a^	FaDu-RR^b^	P value^c^	2A3^d^	P value^c^	DNA damage signaling pathways
*APEX1*			1.89	0.001	BER
*ATM*			1.87	0.01	ATM/ATR signaling, DSB repair, apoptosis, cell cycle
*BAX*			−2.49	0.006	apoptosis
*BBC3*			−3.65	0.003	apoptosis
*BLM*	−1.69	0.02	−7.86	0.000	DSB repair
*BRIP1*			−2.35	0.02	Other repair genes
*CDC25A*			−1.59	0.006	ATM/ATR signaling, cell cycle
*CHEK2*			−1.65	0.02	ATM/ATR signaling, other repair genes, apoptosis, cell cycle
*CIB1*			−5.60	0.001	Other repair genes, apoptosis
*FANCA*			−2.02	0.001	Other repair genes
*HUS1*			−1.65	0.001	ATM/ATR signaling, DSB repair
*MDC1*			−1.66	0.000	DSB repair, cell cycle
*MLH1*			1.51	0.02	DSB repair, mismatch repair
*MPG*			−1.85	0.04	BER
*NBN*			−1.83	0.02	DSB repair
*NTHL1*			−1.54	0.02	BER
*PNKP*			−2.77	0.000	NER
*RBBP8*			1.53	0.02	ATM/ATR signaling, other DNA repair genes
*RNF168*			−2.77	0.001	ATM/ATR signalling, other DNA repair genes
*RPA1*			−1.70	0.04	NER, DSB repair
*SMC1A*			−1.53	0.005	ATM/ATR signalling, other DNA repair genes
*SUMO1*			−2.73	0.002	Other DNA repair genes
*TP73*			−2.12	0.04	Mismatch repair, apoptosis, cell cycle
*XPA*	2.28	0.004	−1.54	0.03	NER
*XRCC3*			−2.20	0.01	other DNA repair genes

^a^ Gene symbol according to HUGO Gene Nomenclature Committee; ^b^ Fold regulation in gene expression in 5 Gy-irradiated FaDu-RR cells in comparison to 5 Gy-irradiated FaDu cells. Positive fold regulation values indicate gene over-expression, negative fold regulation values indicate gene under-expression; ^c^ The P values were calculated based on a Student’s t-test of the replicate 2^(− ΔΔ CT)^ values for each gene in the 5 Gy-irradiated and non-irradiated cells, and differences in gene expression were considered significant for P values less than 0.05. ^d^ Fold regulation in gene expression in 5 Gy-irradiated 2A3 cells in comparison to 5 Gy-irradiated FaDu cells. *DSB* double-strand break, *BER* base excision repair, *NER* nucleotide excision repair

According to the Qiagen’s classification of DNA damage signalling genes, differentially expressed genes in radioresistant FaDu-RR cells were involved in DSB repair and NER. On the other hand, differentially expressed genes in 5 Gy-irradiated 2A3 cells were involved in DSB repair, ATM/ATR signalling, NER, base excision repair, other repair, apoptosis and cell cycle. Functional analysis associated the differentially expressed genes *BLM* and *XPA* in 5 Gy-irradiated FaDu-RR cells with DNA secondary structure binding, single-stranded DNA binding, DNA catabolic process, and structure-specific DNA binding (Additional file 14: Figure S9a). No known interaction networks between *BLM* and *XPA* genes were identified (Additional file 14: Figure S9b). Differentially expressed genes in 5 Gy-irradiated 2A3 cells were associated with DSB repair, DNA catabolic process, DNA damage checkpoint, cell cycle checkpoint, DNA integrity checkpoint, damaged DNA binding, and DNA recombination (Additional file 14: Figure S9c). Using GeneMANIA physical and predicted interactions, pathway, co-expression and co-localization networks among these differentially expressed genes were identified (Additional file 14: Figure S9d).

## Discussion

In order to modulate cellular radioresistance and sensitivity, mechanisms responsible for these phenomena need to be identified. Isogenic cell lines with different radiosensitivity can serve as a good predictive model, as survival changes can be directly attributed to specific modifications in the intrinsic cellular features. Pairs of isogenic cell lines with different radiosensitivity have been established from different solid tumour cell lines, such as head and neck SCC [11, 19–24], prostate carcinoma [25, 26], non-small cell lung cancer [27, 28]. We successfully established radioresistant FaDu-RR cells from parental FaDu cells by repeated exposure to irradiation, while radiosensitive 2A3 cells were derived from parental FaDu cells by stable transfection with HPV16 oncogenes E6 and E7 [12]. Although variations in radiosensitivity of HPV-positive cells exist, they are generally more radiosensitive than the HPV-negative cells [3, 4]. Radiosensitivity of 2A3 cells was confirmed in vivo in experimentally induced tumours in immunodeficient mice [5]. To the best of our knowledge, this is the only study comparing both radioresistant and radiosensitive isogenic cells to parental cells, with a specific focus on gene expression in response to irradiation.

Radioresistant FaDu-RR cells were established by repeated exposure to daily 2 Gy doses from parental FaDu cells, making them 1.6-fold more radioresistant than the parental cells. The observed potentiation of radioresistance in FaDu-RR cells is comparable to the potentiation of radioresistance observed in other isogenic models of radioresistance [23, 28, 29]. The induced radioresistance was either long-term, more than 3 years of passaging [20, 21], or short-term, from 4 weeks to 18 weeks [22, 25, 28–30], however, it could be restored after additional irradiation [30, 31]. FaDu-RR displayed long-term radioresistance of more than 4 years. Protocols used in these studies varied significantly in specific parameters, such as dose/fraction (from 0.5 Gy to 10 Gy), total dose received (from 10 Gy up to more than 1600 Gy), overall treatment time (from 5 days up to 6 years), recovery periods (12 h, daily, weekly, fortnightly intervals) [20, 26, 32, 33]. On the other hand, the recovery period was not always defined in time units, but in the percentage of culture confluence, ranging from 50 to 80% prior to the next fraction [24, 34]. These parameters were determined experimentally, based on the radiosensitivity of the parental cells, and allowing selection of radioresistant clones with replicative potential. In our preliminary study, 10 Gy/fraction with 48 h recovery between fractions was too high for the recovery of radioresistant cells with replicative potential. This was supported by the observation that dose per fraction ranging from 2 to 6 Gy, was sufficient for a selection of radioresistant clones, while lower or higher doses were not [33]. However, others have used lower or higher doses/fraction from 0.5 to 10 Gy combined with shorter recovery period for low dose/fraction, and longer for high dose/fraction to successfully establish radioresistant cells [20, 32]. The observed survival advantages were also inconsistently reported in terms of increased surviving fraction at a specific dose of radiation [11, 19, 22, 23, 25, 29, 32], the dose modifying factor at 2 Gy irradiation dose [28], clonogenic potential [27], mean inactivation dose ratio [28, 35], the dose required to reduce cell survival to 10% [33], and α/β ratio of LQ model [23]. Variations in parameters reporting the survival advantage of radioresistant cells also limit direct comparison of the extent of induced radioresistance. Significant increase in radioresistance can be achieved with either 10 Gy or 1600 Gy of the total dose [26, 32], however, the potentiation of radioresistance cannot be directly compared due to variations in parameters measuring the survival advantages in these cells. In addition to variabilities in protocols used for selection of radioresistant cells, a successful selection of radioresistant cells is dependent also on the cell type [9].

Prolonged exposure to irradiation can select the more radioresistant cells within the cell population, such as cells expressing markers of cancer stem cells (CSC), commonly associated with increased radioresistance [19]. On the other hand, radiation induced genetic instability can further select the more radioresistant cells [9]. During the establishment of radioresistant cells in our study, we have first selected different subclones. By further irradiation, we were not able to select different subclones, but rather whole cell populations. The increased radioresistance observed in the established FaDu-RR cells is a combination of clonal selection of radioresistant cells within the existing cell population and radiation-induced genetic variability as confirmed by differential gene expression.

Changes in the cell cycle distribution can confer different radiosensitivity of cells due to variations in radiosensitivity of cell cycle phases - cells in S-phase being the most radioresistant. We did not observe any difference in cell cycle distribution and cell proliferation rate in non-irradiated isogenic cells. Similarly, no change in the cell cycle distribution or growth rate was observed in other isogenic models of radioresistance [11, 22, 23, 27, 33, 36, 37]. However, 5 h after 5 Gy irradiation, we observed a decrease in G_1_-phase cells and an increase in S-phase and G_2_/M-phase cells with no significant difference between the studied isogenic cells. Similarly, no change in cell cycle distribution was observed in radioresistant and parental cells 4 h after 2 Gy irradiation [25]. While the percent of G_2_/M-phase parental and radioresistant cells remained unchanged 24 h after irradiation, the percent of radiosensitive cells in G_2_/M phase significantly increased. Other reports show a similar level of G_2_/M arrest in response to irradiation in other radiosensitive cells [3, 4, 24]. Increase in G_2_/M-phase cells was observed both in radiosensitive and radioresistant cells, but the disturbance of the cell cycle in response to irradiation was longer in radiosensitive cells [38]. Based on these results, other mechanisms than alterations in cell cycle regulation were involved in the radioresistant phenotype of FaDu-RR cells.

Alterations in radiation-induced apoptosis can also contribute to altered cellular radiosensitivity. In our study, no apoptosis was observed in non-irradiated isogenic cells and none of the differentially expressed genes observed in the non-irradiated radioresistant cells were associated with the apoptotic processes. On the other hand, the proapoptotic *BBC3* gene was under-expressed in 2A3 radiosensitive cells. The reason for this could be a transfection of radiosensitive cells by HPV protein E6 [12], which is known to interact with pro-apoptotic proteins to prevent apoptosis in HPV-positive cells [39, 40]. In response to 5 Gy irradiation, fold-induction of apoptosis was the highest in parental cells, and only non-significantly increased in the radioresistant cells 72 h after irradiation. In addition, proapoptotic *BBC3* was under-expressed in radioresistant cells in response to irradiation. This indicates the involvement of anti-apoptotic regulatory mechanisms in radioresistant cells. Variability in the induction of apoptosis in response to radiation exists likely due to different radiation dose, time after irradiation, and the method used to detect specific hallmarks of apoptosis. Similarly, in the study by Wei QC et al., no induction of apoptosis or apoptotic morphological features were observed up to 48 h after 6 Gy irradiation in radioresistant and parental cells [28]. Contrary, 24 h after 10 Gy irradiation less apoptotic cells were detected in the radioresistant population [27].

Cell survival after irradiation largely depends on the balance between DNA damage signalling, induction, and repair. We evaluated DNA damage by immunofluorescence staining of γH2AX foci, surrogate markers of DNA DSBs [41]. We observed no significant difference in endogenous levels of γH2AX foci in the studied isogenic cells. However, the range of the observed γH2AX foci levels was the highest in the 2A3 radiosensitive cells. More endogenous DNA damage was expected in the cells due to E6-mediated oxidative stress [42]. On the other hand, some studies reported radioresistant cells to have less endogenous DNA damage, more effective DNA repair, and enhanced levels of ROS scavengers [19, 23–25, 27, 28]*.*

After irradiation, the induction of γH2AX foci was the most prominent in radiosensitive cells, while in radioresistant cells the induction of foci was delayed. In addition, at the peak of γH2AX foci expression, radioresistant cells displayed the lowest number of γH2AX foci/nucleus in contrast to the highest number of γH2AX foci/nucleus observed in radiosensitive cells. The highest percent of γH2AX-positive cells was observed in radiosensitive cells and the lowest in radioresistant cells. As expected, the resolution of γH2AX foci was the most prominent in radioresistant cells. Similarly to our results, less γH2AX foci/nucleus were observed in radioresistant cells in comparison to radiosensitive cells after irradiation [43]. Loss of γH2AX foci was the most prominent in radioresistant cells, indicating a greater DNA DSB repair as another alteration contributing to radioresistant phenotype [44].

DNA DSB is the most lethal lesion induced by irradiation, and if not repaired, leads to cell death. Biphasic kinetics of DNA DSB repair reflects the mechanistic differences of the two major repair pathways, homologous recombination (HR) and non-homologous end-joining (NHEJ) [45]. NHEJ is a fast, highly efficient, error-prone process active throughout the cell cycle, but predominantly in G_1_-phase cells. On the other hand, HR is a slow, accurate process active in S- and G_2_-phase cells in the presence of sister chromatid. In our study, radiosensitive cells displayed initial fast repair, followed by a slow resolution of γH2AX foci, while the radioresistant cells exhibited fast γH2AX foci resolution in both phases of DSB repair. Similar to our study, faster disappearance of γH2AX was observed in other radioresistant cells compared to slower γH2AX foci resolution in radiosensitive cells [38, 46]. We concluded that the higher ability to repair DSB observed in studied radioresistant cells, in comparison to both parental and radiosensitive cells by both NHEJ and HR, importantly contributes to the cellular radioresistance.

Incorrect rejoining of radiation-induced DNA breaks can lead to structural and numerical chromosomal alterations [47]. As observed by flow cytometry isogenic FaDu, FaDu-RR, and 2A3 cells do not differ in chromosome number. On the other hand, alterations in chromosome structure can affect gene expression. A more detailed genomic analysis is needed to specifically identify radiation-induced mutations, loss and gain of genetic material, and chromosomal abberations. The observed under-expression and over-expression of DNA DSR genes could be either due to alterations of the genetic material, or due to changes in the translation of DNA.

We further focused on DNA DSR gene expression because alterations of these genes can importantly determine radiosensitivity [48]. We observed alterations in the expression of studied genes in non-irradiated radioresistant and radiosensitive cells, compared with parental cells. We also observed over-expression of DNA DSR gene expression in parental and radioresistant cells, but not in radiosensitive cells 5 h after irradiation. Similarly, other studies found more DNA repair genes to be up-regulated in radioresistant cells 6 h after irradiation than in radiosensitive cells [49]. Prolonged transcriptional activity of DNA damage response, cell cycle and apoptosis-related genes was observed in radiosensitive cells in comparison to radioresistant cells in response to irradiation [38].

We identified 4 genes of interest, *FANCD2*, *GADD45A*, *H2AFX*, and *XRCC2* to be over-expressed in radiosensitive cells, but under-expressed in radioresistant cells. *FANCD2* gene product is an important component of the Fanconi anemia pathway of DNA damage response and acts as a recruitment factor for other DNA repair proteins [50]. *FANCD2* accumulation is associated with HPV-activated Fanconi anemia pathway and is essential for the maintenance of viral episomes in HPV-infected cells [51]. Over-expression of *FANCD2* in radiosensitive 2A3 cells could be associated with the HPV16 E6 and E7 transfection.

*GADD45A* gene product is involved in cell cycle regulation, DNA repair, and apoptosis [52]. Over-expression of *GADD45A* promotes transcriptional activity through global demethylation [53, 54]. Based on these results, the detected over-expression of *GADD45A* in radiosensitive cells could be associated with the increased transcriptional activity and differential gene expression observed in these cells in comparison to parental cells. On the other hand, silencing *GADD45A* is associated with increased survival, reduced apoptosis, and reduced sensitivity to CDDP [54, 55]. Under-expression of *GADD45A* could contribute to CDDP cross-resistance in radioresistant FaDu-RR cells. These data suggest a radioprotective mechanism through under-expression of *GADD45A*. However, contrary to these observations, under-expression of *GADD45A* in melanoma cells increased sensitivity to CDDP and enhanced CDDP-induced DNA damage [56]. In addition, *GADD45A* gene and protein expression are increased in response to a variety of DNA damaging agents, including ionizing radiation [52, 55, 57]. Similarly, we observed an increase in *GADD45A* gene expression 5 h after irradiation in parental and radioresistant cells, but not in radiosensitive cells. It is possible that intrinsically higher levels of *GADD45A* in radiosensitive cells limit any further increase in the expression of this gene in response to irradiation. The observed over-expression of genes in parental and radioresistant cells, but not in radiosensitive cells, could be associated with *GADD45A*-mediated increase in transcriptional activity [53, 54].

*H2AFX* gene encodes histone H2AX, which is rapidly phosphorylated to γH2AX in response to DNA DSBs induced by radiation or DNA damaging agents. γH2AX then interacts with MDC1, another over-expressed gene in radioresistant cells in response to irradiation, and recruits other DNA damage response-associated proteins to the site of the DSB [16]. Silencing *H2AX* induced activation of epithelial-mesenchymal transition factors, and promoted metastatic behavior [58]. Over-expression of H2AFX observed in radiosensitive cells is in agreement with the observed higher range of γH2AX foci observed by immunofluorescence. On the other hand, over-expression of *H2AFX* in radioresistant cells, but not parental or radiosensitive cells, in response to irradiation could be an indication of enhanced recognition of DSBs, and faster disappearance of γH2AX foci observed.

*XRCC2* gene product is a paralogue of RAD51 protein, involved in DNA DSB repair via HR [59]. Knockdown of *XRCC2* in colon carcinoma cells decreased cell proliferation, increased apoptosis and lead to cell cycle arrest induced by irradiation [60]. XRCC2 is also involved in the regulation of replication fork progression to prevent DNA damage and genomic instability [61]. Although *XRCC2* is under-expressed in radioresistant cells, its expression is up-regulated early in the response to 5 Gy irradiation, which could contribute to faster DSB repair by HR in radioresistant cells. No change in *XRCC2* expression in parental and radiosensitive cells was observed in response to irradiation which is in agreement with slower HR repair of DNA DSB.

Another Rad51 paralogue, *XRCC3* was the only under-expressed gene in radiosensitive cells in response to the irradiation, indicating on a possible mechanism of increased sensitivity to ionizing radiation. As shown by Cheng J et al, knockdown of *XRCC3* increased radiosensitivity in vitro and in vivo, while high expression of *XRCC3* correlated with radio- and chemoresistance [62].

By direct comparison of gene expression in 5 Gy-irradiated isogenic cells, we identified another gene of interest, *XPA*, possibly involved in the radioresistance mechanism. *XPA* gene product plays a central role in the NER pathway, through which CDDP-DNA adducts are repaired [63]. *XPA* was over-expressed in radioresistant cells, but under-expressed in radiosensitive cells in response to irradiation. This was also observed in radioresistant glioblastoma cells [64]. The increased expression of *XPA*, and consequently activated NER could be associated with cross-resistance to CDDP, observed in radioresistant cells. In a parallel study, we demonstrated a more efficient repair of CDDP and BLM-induced DNA damage in radioresistant cells compared to parental cells [15]. Other mechanisms contributing to CDDP resistance include an impaired influx of CDDP, and activation of different multidrug mechanisms, including glutathione and multidrug-resistance associated protein (MRP) [30, 65]. Alterations in sensitivity to specific cytotoxic agents, including increased resistance and sensitivity, were observed in many experimentally induced radioresistant cells [21, 22, 25, 27, 28, 30]. Increased level of ROS scavengers associated with cellular radioresistance can also reduce sensitivity to platinum drugs through neutralization of platinum-induced oxidative stress [24, 27, 66]. On the other hand, the isogenic radiosensitive cells were more sensitive to CDDP, Oxa, and BLM. Isogenic pairs of HPV-positive and HPV-negative cells have not been evaluated in terms of chemosensitivity and the available data on CDDP sensitivity of HPV-positive head and neck SCC is not conclusive. HPV-positive cells were found to be either CDDP-resistant [67] or equally sensitive to CDDP compared to HPV-negative head and neck SCC [68].

The observed differences in DNA damage signaling gene expression in isogenic cells with different radiosensitivity indicate a role of several DNA damage signalling genes in the mechanisms of radioresistance. However, this should be further confirmed by quantitative and functional expression of specific proteins which is one of the future perspectives of this study. Identification of deregulated proteins could be applied clinically in terms of either radioresistance-associated biomarkers or as potential targets for radiosensitization. These novel biomarkers related to either radiosensitivity or radioresistance would permit improved stratification of HNSCC patients based on the predicted response to irradiation [69]. Radiosensitization of more radioresistant tumors could also lead to reduced total irradiation dose, fewer and/or milder adverse effects. Dose de-escalation is a feasible approach for HPV-positive oropharyngeal HNSCC, with high response rate and reduced toxicity [5, 70, 71].

## Conclusion

This study showed that experimentally induced radioresistant cells are a suitable model to study the mechanisms of radioresistance conferring survival advantage. The experimentally induced radioresistance in our isogenic model is primarily associated with lower susceptibility to DNA damage, faster DNA repair and CDDP cross-resistance. The results were confirmed by both functional assays and transcriptomic analysis of DNA DSR genes. Furthermore, we identified several deregulated genes likely to play a role in the radioresistance and potential targets to achieve cellular radiosensitization. In addition, our results indicate a lack of adaptive response to irradiation in radiosensitive cells, possibly due to reduced ability to repair a high number of DNA DSBs via HR as demonstrated by immunofluorescence staining of γH2AX foci and differential gene expression in response to irradiation. To confirm their role in radioresistance, further experiments, beyond the scope of this study are needed. We propose targeting specific genes *XRCC2*, *XRCC3*, *H2AFX,* and *GADD45A*. In order to confirm their role in radiomodulation, enhancing their expression in radioresistant cells, or silencing them in radiosensitive or parental cells should be tested. Additionally, to avoid cross-resistance to CDDP in radioresistant cells enhancing expression of *GADD45A* or silencing of *XPA* could increase sensitivity to CDDP.

## Supplementary information

**Additional file 1.** Final report of laboratory examination.

**Additional file 2.** Final report of laboratory examination.

**Additional file 3: Figure S1.** Representative DNA content frequency histograms of (**a**) isogenic parental FaDu, (**b**) radioresistant FaDu-RR, and (**c**) radiosensitive 2A3 cells before irradiation, 5 h and 24 h after 5 Gy irradiation.

**Additional file 4: Figure S2.** Representative images of flow cytometry detection of apoptosis by FITC Annexin V/7-AAD staining before irradiation, 5 h, 24 h, 48 h and 72 h after 5 Gy irradiation. Bottom left quadrant are viable (Annexin V-negative and 7AAD-negative) cells, bottom right quadrant are early apoptotic (Annexin V-positive and 7AAD-negative) cells, top right quadrant are late apoptotic and/or necrotic (Annexin V-positive and 7AAD-positive) cells, and top left quadrant are necrotic (Annexin V-negative and 7AAD-positive) cells.

**Additional file 5: Table S1.** A list of genes included on RT^2^ Profiler™ PCR Array Human DNA Damage Signaling Pathway (PAHS-029Z, Qiagen).

**Additional file 6: Table S2.** Radiosensitivity parameters describing survival advantage of isogenic head and neck SCC cells with different radiosensitivity.

**Additional file 7: Figure S3.** Changes in cell cycle phase distribution at different time points before and after 5 Gy irradiation in isogenic parental FaDu, radioresistant FaDu-RR and radiosensitive 2A3 cells. Symbols are mean values with sem from three independent experiments. * indicates significant difference in comparison to non-irradiated control cells; n.s. – non-significant.

**Additional file 8: Table S3.** Percentage of early and late apoptotic cells in control non-irradiated isogenic cells.

**Additional file 9: Figure S4.** Fold-induction of apoptosis in parental FaDu (a), radioresistant FaDu-RR (b) and radiosensitive 2A3 cells (c) at different time points after 5 Gy irradiation. Symbols are mean values with sem from three independent experiments. * indicates significant difference between control and 5 Gy-irradiated cells; ** indicated significant difference between 5 h and 72 h; # indicates significantly increased fold-induction of apoptosis; n.s. – non-significant.

**Additional file 10: Figure S5.** Fold-change in cell viability of isogenic parental FaDu (a), radioresistant FaDu-RR (b) and radiosensitive 2A3 cells (c) at different time points after 5 Gy irradiation. Symbols are mean values with sem from three independent experiments. *** indicates significantly different fold-change of cell viability in comparison to non-irradiated cells and 5 Gy irradiated FaDu cells at earlier time points; * indicates significantly different fold-change in cell viability between non-irradiated and 5 Gy irradiated FaDu-RR cells 72 h after irradiation; ** indicates significantly different fold-change of cell viability in comparison to non-irradiated and 5 Gy irradiated 2A3 cells 5 h and 24 h after irradiation; # indicates significantly different fold-change of cell viability in comparison to non-irradiated 2A3 cells 24 h, 48 h and 72 h.

**Additional file 11: Figure S6. (a)** Normalized nuclei area, **(b)** percent of enlarged nuclei, and **(c)** median number of γH2AX foci/nuclei in parental FaDu, radioresistant FaDu-RR, and radiosensitive 2A3 cells at various recovery times after 5 Gy irradiation. Box plots are median with 25th and 75th percentile with 10th and 90th percentiles as bottom and top whiskers. Bars are pooled data from three independent experiments. * indicates significantly enlarged normalized nuclei area compared to other groups.

**Additional file 12: Figure S7.** Functional analysis of DNA damage signalling and repair gene expression in non-irradiated isogenic cells. (**a**) Top 7 functions associated with the differentially expressed genes in radioresistant FaDu-RR cells. (**b**) Interactions between the differentially expressed genes in radioresistant FaDu-RR cells. (**c**) Top 7 functions associated with the differentially expressed genes in radiosensitive 2A3 cells. (**d**) Interactions between the differentially expressed genes in radiosensitive 2A3 cells. Functions of and interactions between the genes were visualized through GeneMANIA. Circles represent differentially expressed genes in comparison to non-irradiated parental FaDu cells. The colours within the circle represent specific functions associated with the specific gene. The coloured links between the genes represent the type of interaction between the differentially expressed genes. (**e**) Schematic diagram of differentially expressed genes involved in specific over-represented pathways of DNA repair, cell cycle and programmed cell death in non-irradiated radioresistant FaDu-RR (green box) and radiosensitive 2A3 cells (blue box). Genes in bold are differentially expressed in both radioresistant FaDu-RR and radiosensitive 2A3 cells. An upward pointing arrow indicates over-expression, a downward pointing arrow indicates under-expression of a specific gene. A schematic diagram is adapted from Reactome Pathway Database.

**Additional file 13: Figure S8.** Functional analysis of DNA damage signalling and repair gene expression in irradiated isogenic cells. (**a**) Top 7 functions associated with the differentially expressed genes in 5 Gy-irradiated parental FaDu cells. (**b**) Interactions between the differentially expressed genes in 5 Gy-irradiated parental FaDu cells. (**c**) Top 7 functions associated with the differentially expressed genes in 5 Gy-irradiated radioresistant FaDu-RR cells. (**d**) Interactions between the differentially expressed genes in 5 Gy-irradiated radioresistant FaDu-RR cells. Functions of and interactions between the genes were visualized through GeneMANIA. Circles represent differentially expressed genes in 5 Gy-irradiated cells relative to pertinent non-irradiated cells. The colors within the circle represent specific functions associated with the specific gene. The colored links between the genes represent the type of interaction between the differentially expressed genes. (**e**) Schematic diagram of differentially expressed genes involved in specific pathways of DNA repair, cell cycle and programmed cell death in 5 Gy-irradiated parental FaDu (light blue box), radioresistant FaDu-RR (green box) and radiosensitive 2A3 cells (blue box). Genes in bold are differentially expressed in both parental FaDu and radioresistant FaDu-RR cells. An upward pointing arrow indicates over-expression; a downward pointing arrow indicates under-expression of a specific gene. A schematic diagram is adapted from Reactome Pathway Database.

**Additional file 14: Figure S9.** Functional analysis of DNA damage signalling and repair gene expression in irradiated isogenic cells. (**a**) Top 7 functions associated with the differentially expressed genes in 5 Gy-irradiated radioresistant FaDu-RR cells. (**b**) Interactions between the differentially expressed genes in 5 Gy-irradiated radioresistant FaDu-RR cells. (**c**) Top 7 functions associated with the differentially expressed genes in 5 Gy-irradiated radiosensitive 2A3 cells. (**d**) Interactions between the differentially expressed genes in 5 Gy-irradiated radiosensitive 2A3 cells. Functions of and interactions between the genes were visualized through GeneMANIA. Circles represent differentially expressed genes in comparison to 5 Gy-irradiated parental FaDu cells. Colours within the circle represent specific functions associated with the specific gene. The coloured links between the genes represent the type of interaction between the differentially expressed genes. (**e**) Schematic diagram of differentially expressed genes involved in specific pathways of DNA repair, cell cycle and programmed cell death in 5 Gy-irradiated radioresistant FaDu-RR (green box) and radiosensitive 2A3 cells (blue box). Genes in bold are differentially expressed in both radioresistant FaDu-RR and radiosensitive 2A3 cells. An upward pointing arrow indicates over-expression, a downward pointing arrow indicates under-expression of a specific gene. A schematic diagram is adapted from Reactome Pathway Database.

## Data Availability

The datasets during and/or analysed during the current study available from the corresponding author on reasonable request.

## Abbreviations

AM: Arithmetic mean
BER: Base excision repair
BLM: Bleomycin
CDDP: Cisplatin
D_10_: Dose required to kill 90% of the cells
DMF: Dose-modifying factor
DSB: Double-strand break
DSR: Damage signalling and repair
DT: Doubling time
ED_50_: Effective dose killing 50% of the cells
HPV: Human papillomavirus
HR: Homologous recombination
IC_50_: Half-maximal inhibitory concentration
LQ: Linear-quadratic model
NER: Nucleotide excision repair
NHEJ: Non-homologous end-joining
Oxa: Oxaliplatin
ROS: Reactive oxygen species
RT-PCR: Real time reverse transcription polymerase chain reaction
SCC: Squamous cell carcinoma
SEM: Standard error of the mean
SF2: Surviving fraction at 2 Gy
