# Supplementary material for: Mechanisms of different response to ionizing irradiation in isogenic head and neck cancer cell lines
Source: Radiat Oncol. 2019 Nov 27;14:214. doi: 10.1186/s13014-019-1418-6 (PMC6882348; doi:10.1186/s13014-019-1418-6)
Supplement: Supplementary file 1 — Additional file 1. Final report of laboratory examination. [file 13014_2019_1418_MOESM1_ESM.pdf]

IDEXX BioAnalytics

FINAL REPORT OF LABORATORY EXAMINATION

Mörikestr. 28/3, D 71636 Ludwigsburg, Germany
+49 (0)7141 64 83585
idexxbioanalytics-europe@idexx.com www.idexxbioanalytics.eu/

IDEXX BioAnalytics Case # 63647-2019

Received: 7/23/2019
Completed: 7/31/2019

Submitted By

Vesna Todorovic
Institute of Oncology Ljubljana
Department of Experimental Oncology
Zaloska 2
Ljubljana SI-1000
Slovenia

Phone: 38615879437
Email: vtodorovic@onko-i.si; Idexx-radil-results@idexx.com

Specimen Description

Species: human
Description: Cells
Number of Specimens/Animals: 2

ID	Client ID	Species	ATCC #	OTHER 1	Specimen
1	FaDu	human	HTB-43		cell
2	FaDu-RR	human		established from FaDu (HTB-43)	cell

Services/Tests Performed: CellCheck 16 - human (16 Marker STR Profile and Inter-species Contamination Test) (1-2)

Genetic evaluation for: Human 16-Marker STR Profile, Interspecies Contamination Test

Summary: Cell Check results are provided in the data results section for each sample. For human samples, an identity matching score above 80% indicates the sample is consistent with the cell line of origin. For human samples with less than an 80% matching score, please see individual comments for these samples in the detail section.

Please see the report for details.

CELL CHECK

Species-specific PCR Evaluation

Species	1	2
mouse	-	-
rat	-	-
human	+	+
Chinese hamster	-	-
African green monkey	-	-

Marker Analysis

Marker Name	1		2	
	Sample Results	FaDu (Nature 520:307-311, 2015)	Sample Results	FaDu (Nature 520:307-311, 2015)
AMEL	NA	NA	NA	NA
CSF1PO	12	12	12	12
D13S317	8, 9	8, 9	8, 9	8, 9
D16S539	11	11	11	11
D18S51	16	16	16	16
D21S11	31.2	31.2	31.2	31.2
D3S1358	17, 18	17, 18	17	17, 18
D5S818	12	12	12	12
D7S820	11, 12	11, 12	11, 12	11, 12
D8S1179	13	13	13	13
FGA	25	25	25	25
Penta_D	11	11	11	11
Penta_E	17, 19	17, 19	19	17, 19
TH01	8	8	8	8
TPOX	11	11	11	11
vWA	15, 17	15, 17	15, 17	15, 17
Identity Match	100%		> 80%	
