# Supplementary material for: Mechanisms of different response to ionizing irradiation in isogenic head and neck cancer cell lines
Source: Radiat Oncol. 2019 Nov 27;14:214. doi: 10.1186/s13014-019-1418-6 (PMC6882348; doi:10.1186/s13014-019-1418-6)
Supplement: Supplementary file 2 — Additional file 2. Final report of laboratory examination. [file 13014_2019_1418_MOESM2_ESM.pdf]

IDEXX BioAnalytics

FINAL REPORT OF LABORATORY EXAMINATION

Mörikestr. 28/3, D 71636 Ludwigsburg, Germany
+49 (0)7141 64 83585
idexxbioanalytics-europe@idexx.com www.idexxbioanalytics.eu/

IDEXX BioAnalytics Case # 63645-2019

Received: 7/23/2019
Completed: 7/31/2019

Submitted By

Vesna Todorovic
Institute of Oncology Ljubljana
Department of Experimental Oncology
Zaloska 2
Ljubljana SI-1000
Slovenia

Phone: 38615879437
Email: vtodorovic@onko-i.si; Idexx-radil-results@idexx.com

Specimen Description

Species: human
Description: Cells
Number of Specimens/Animals: 11

ID	Client ID	Cell Line	Species	ATCC #	Other 1	Specimen
1	2A3	2A3	human	CRL-3212	(HPV) DNA sequences	cell
2	HepG2	HepG2	human	HB-8065		cell
3	SKMEL-28	SKMEL-28	human	HTB-72		cell
4	PC3	PC3	human	CRL-1435		cell
5	DU-145	DU-145	human	HTB-81		cell
6	Caco-2	Caco-2	human	HTB-37		cell
7	HT29-MTX-E12	HT29-MTX-E12	human		ECACC 12040401	cell
8	LoVo	LoVo	human	CCL-229		cell
9	Wi-38	Wi-38	human	CCL-75		cell
10	HUVEC	HUVEC	human	CRL-1730		cell
11	HMEC-1	HMEC-1	human	CRL-3243	Biosafety level 2 (SV-40)	cell

Services/Tests Performed: CellCheck 9 - human (9 Marker STR Profile and Inter-species Contamination Test) (1-11)

Genetic evaluation for: Human 9-Marker STR Profile, Interspecies Contamination Test

Summary: Cell Check results are provided in the data results section for each sample. For human samples, an identity matching score above 80% indicates the sample is consistent with the cell line of origin. For human samples with less than an 80% matching score, please see individual comments for these samples in the detail section.

Please see the report for details.

CELL CHECK

Species-specific PCR Evaluation

Species	1	2	3	4	5	6	7	8	9	10
mouse	-	-	-	-	-	-	-	-	-	-
rat	-	-	-	-	-	-	-	-	-	-
human	+	+	+	+	+	+	+	+	+	+
Chinese hamster	-	-	-	-	-	-	-	-	-	-
African green monkey	-	-	-	-	-	-	-	-	-	-

Species	11
mouse	-
rat	-
human	+
Chinese hamster	-
African green monkey	-

Marker Analysis

Marker Name	1		2		3		4		5		6	
	Sample Results	2A3 (ATCC# CRL-3212)	Sample Results	Hep G2 (ATCC# HB-8065)	Sample Results	SK-Mel-28 (ATCC# HTB-72)	Sample Results	PC-3 (ATCC# CRL-1435)	Sample Results	DU-145 (ATCC# HTB-81)	Sample Results	CaCo-2 (ATCC# HTB-37)
AMEL	NP	NA	X, Y	X, Y	X, Y	X, Y	X	X	X, Y	X, Y	X	X
CSF1PO	12	12	10, 11	10, 11	10, 12	10, 12	11	11	10, 11	10, 11	11	11
D13S317	8, 9	8, 9	9, 13	9, 13	11, 12	11, 12	11	11	12, 14	12, 13, 14	11, 13, 14	11, 13, 14
D16S539	11	11	12, 13	12, 13	9, 12	9, 12	11	11	11, 12, 13	11, 13	12, 13	12, 13
D5S818	12	12	11, 12	11, 12	11, 13	11, 13	13	13	10, 13	10, 13	12, 13	12, 13
D7S820	11, 12	11, 12	10	10	9.3, 10	9.3, 10	8	8, 11	7, 10, 11	7, 10, 11	11, 12	11, 12
TH01	8	8	9	9	7	7	6, 7	6, 7	7	7	6	6
TPOX	11	11	8, 9	8, 9	8, 12	8, 12	8, 9	8, 9	11	11	9, 11	9, 11
vWA	15, 17	15, 17	17	17	19	16, 19	17	17	17, 18	17, 18, 19	16, 18	16, 18
Identity Match	100%		100%		> 80%		> 80%		> 80%		100%	

Marker Name	7		8		9		10		11	
	Sample Results	HT-29 (ATCC# HTB-38)	Sample Results	LoVo (ATCC# CCL-229)	Sample Results	WI-38 (ATCC# CCL-75)	Sample Results	HUV-EC-C (ATCC# CRL-1730)	Sample Results	HMEC-1 (ATCC# CRL-3243)
AMEL	X	X	X, Y	X, Y	X	X	X	X	X, Y	X, Y
CSF1PO	11, 12	11, 12	11, 13, 14	10, 11, 13, 14	10, 12	10, 12	12	11, 12	10, 12	10, 12
D13S317	11	11, 12	8, 11	8, 11	11	11	12	9, 11	12	11, 12
D16S539	11, 12	11, 12	9, 12	9, 12	11, 12	11, 12	9	11, 12	11, 12	11, 12

Marker Name	7		8		9		10		11	
	Sample Results	HT-29 (ATCC# HTB-38)	Sample Results	LoVo (ATCC# CCL-229)	Sample Results	WI-38 (ATCC# CCL-75)	Sample Results	HUV-EC-C (ATCC# CRL-1730)	Sample Results	HMEC-1 (ATCC# CRL-3243)
D5S818	11, 12	11, 12	11, 13	11, 12, 13	10	10	10	11, 12	12, 13	12
D7S820	10	10	9.3, 10, 11	9.3, 10, 11	9, 11	9, 11	10, 11	8, 12	8, 10	8, 10
TH01	6	6, 9	9.3	9.3	9.3	9.3	6	6, 9.3	7	7
TPOX	8, 9	8, 9	8, 9	8, 9	8	8	8, 11	8, 11	8, 11	8, 11
vWA	17	17, 19	17, 18	17, 18	19, 20	19, 20	17	16	15, 17	15, 17
Identity Match	> 80%		> 80%		100%		< 80%, see comments		> 80%	

Sample ID	Remarks
10	The genetic profile is not consistent with the genetic profile reported for the HUV-EC-C cell line (ATCC# CRL-1730). The sample profile is identical to the genetic profile reported for the ECV-304 cell line (ATCC# CRL-1998) and HBMEC-2 cell line (ATCC# CRL-3023). This sample appears to be misidentified.
