# Supplementary figures and images for: Mechanisms of different response to ionizing irradiation in isogenic head and neck cancer cell lines
Source: Radiat Oncol. 2019 Nov 27;14:214. doi: 10.1186/s13014-019-1418-6 (PMC6882348; doi:10.1186/s13014-019-1418-6)

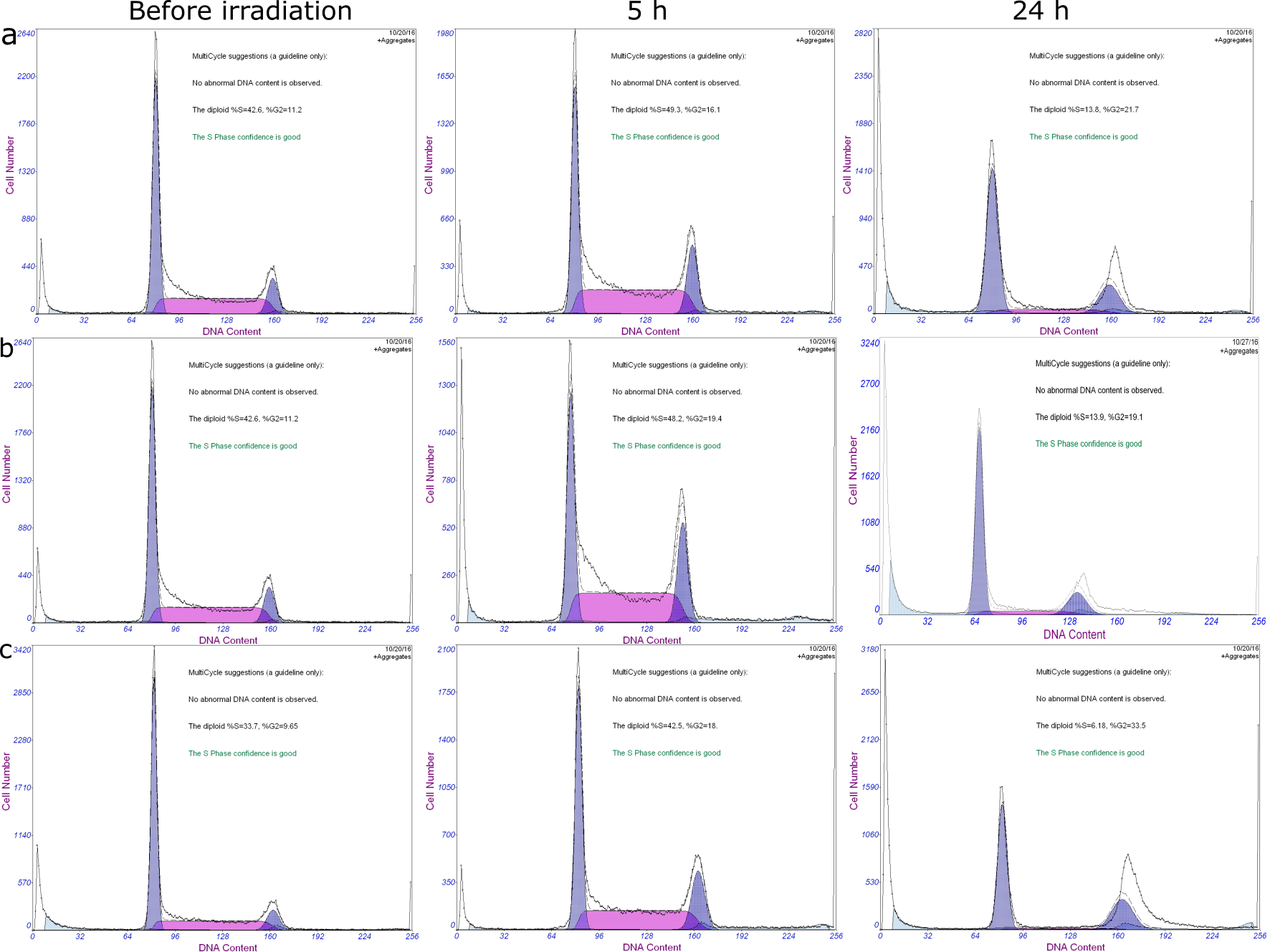

Supplement: Supplementary file 3 — Additional file 3: Figure S1. Representative DNA content frequency histograms of (a) isogenic parental FaDu, (b) radioresistant FaDu-RR, and (c) radiosensitive 2A3 cells before irradiation, 5 h and 24 h after 5 Gy irradiation. [file 13014_2019_1418_MOESM3_ESM.tif]

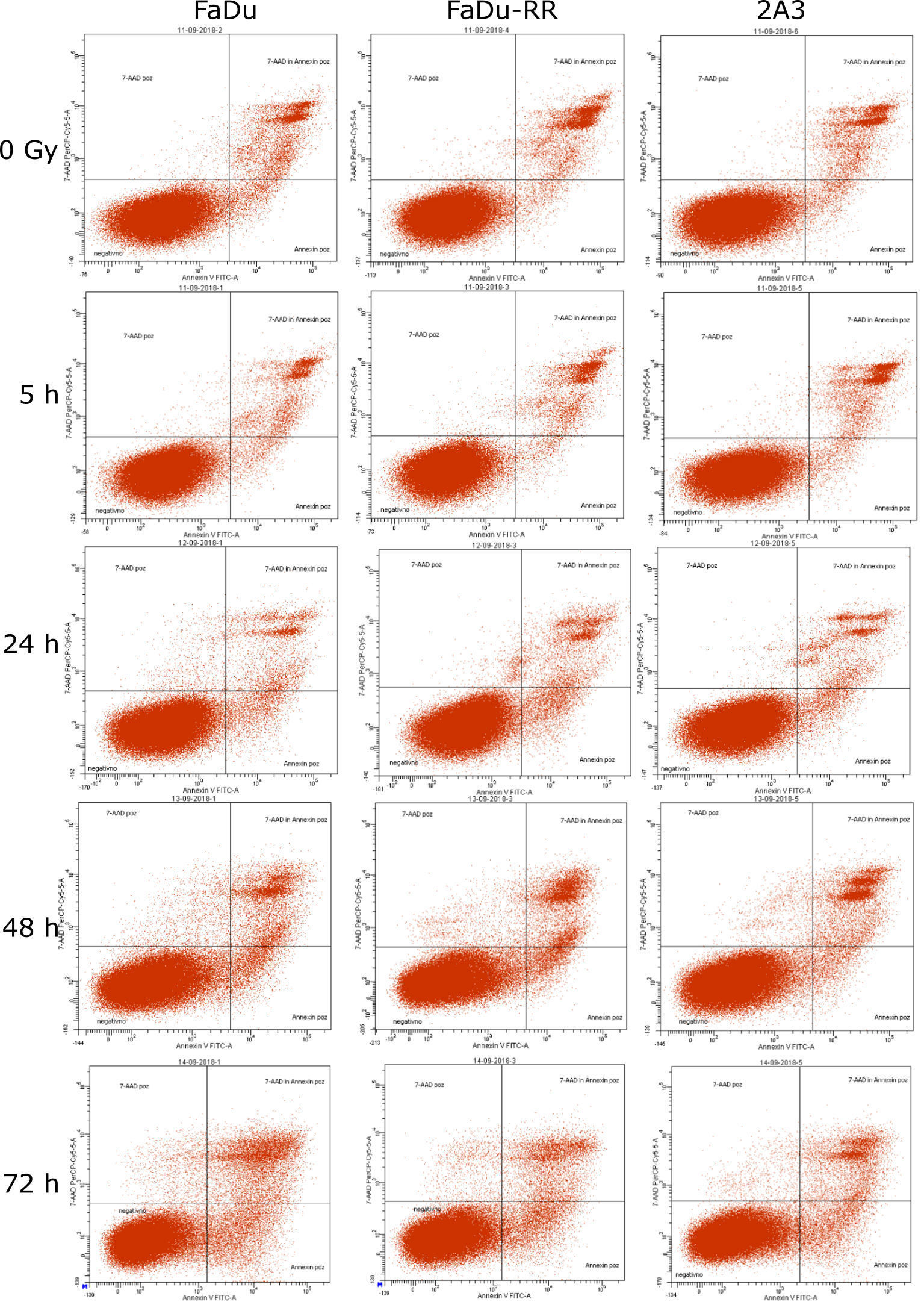

Supplement: Supplementary file 4 — Additional file 4: Figure S2. Representative images of flow cytometry detection of apoptosis by FITC Annexin V/7-AAD staining before irradiation, 5 h, 24 h, 48 h and 72 h after 5 Gy irradiation. Bottom left quadrant are viable (Annexin V-negative and 7AAD-negative) cells, bottom right quadrant are early apoptotic (Annexin V-positive and 7AAD-negative) cells, top right quadrant are late apoptotic and/or necrotic (Annexin V-positive and 7AAD-positive) cells, and top left quadrant are necrotic (Annexin V-negative and 7AAD-positive) cells. [file 13014_2019_1418_MOESM4_ESM.tif]

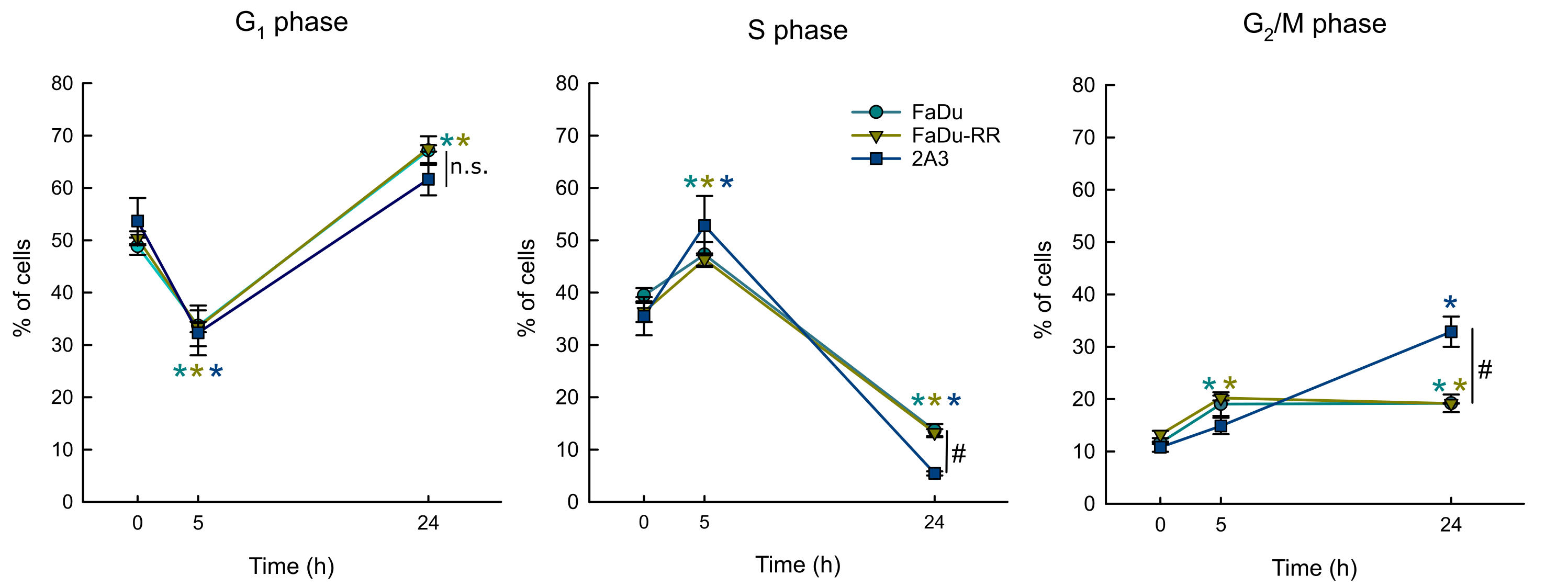

Supplement: Supplementary file 7 — Additional file 7: Figure S3. Changes in cell cycle phase distribution at different time points before and after 5 Gy irradiation in isogenic parental FaDu, radioresistant FaDu-RR and radiosensitive 2A3 cells. Symbols are mean values with sem from three independent experiments. * indicates significant difference in comparison to non-irradiated control cells; n.s. – non-significant. [file 13014_2019_1418_MOESM7_ESM.tif]

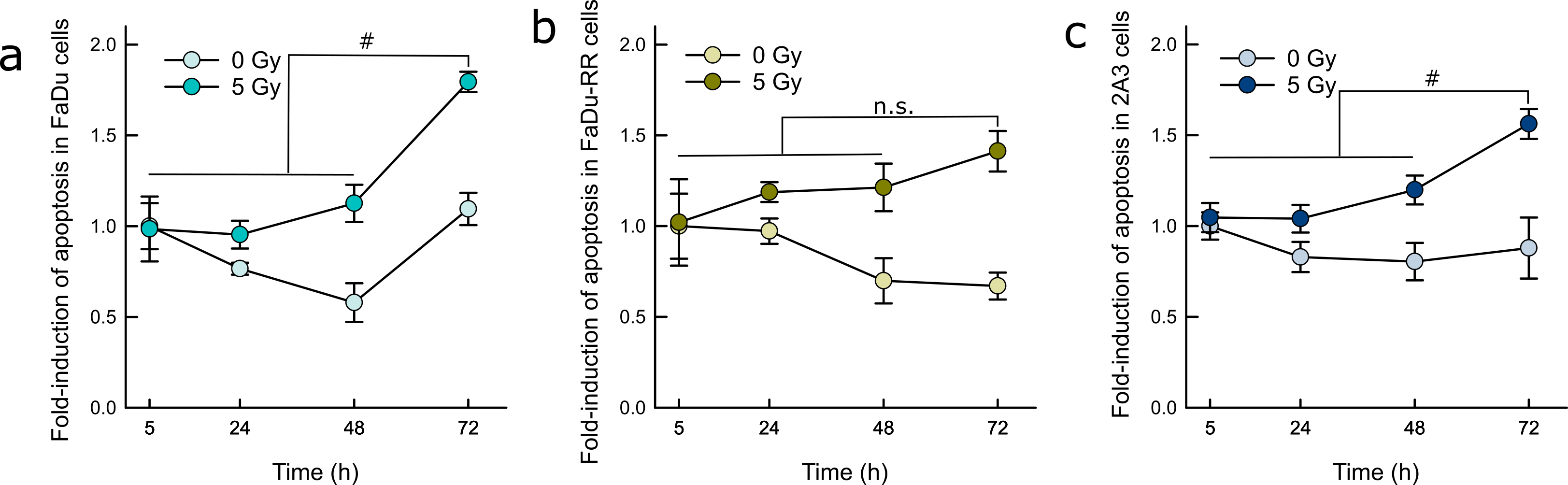

Supplement: Supplementary file 9 — Additional file 9: Figure S4. Fold-induction of apoptosis in parental FaDu (a), radioresistant FaDu-RR (b) and radiosensitive 2A3 cells (c) at different time points after 5 Gy irradiation. Symbols are mean values with sem from three independent experiments. * indicates significant difference between control and 5 Gy-irradiated cells; ** indicated significant difference between 5 h and 72 h; # indicates significantly increased fold-induction of apoptosis; n.s. – non-significant. [file 13014_2019_1418_MOESM9_ESM.tif]

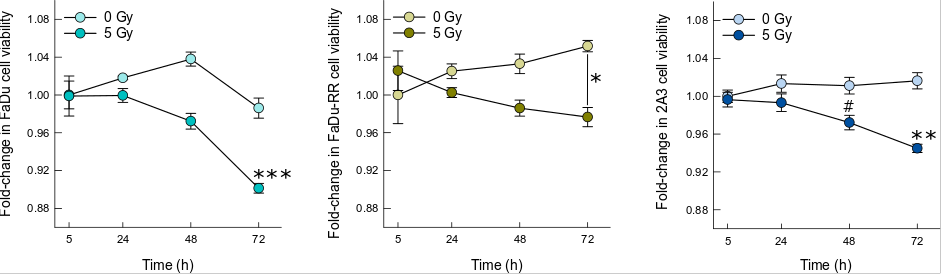

Supplement: Supplementary file 10 — Additional file 10: Figure S5. Fold-change in cell viability of isogenic parental FaDu (a), radioresistant FaDu-RR (b) and radiosensitive 2A3 cells (c) at different time points after 5 Gy irradiation. Symbols are mean values with sem from three independent experiments. *** indicates significantly different fold-change of cell viability in comparison to non-irradiated cells and 5 Gy irradiated FaDu cells at earlier time points; * indicates significantly different fold-change in cell viability between non-irradiated and 5 Gy irradiated FaDu-RR cells 72 h after irradiation; ** indicates significantly different fold-change of cell viability in comparison to non-irradiated and 5 Gy irradiated 2A3 cells 5 h and 24 h after irradiation; # indicates significantly different fold-change of cell viability in comparison to non-irradiated 2A3 cells 24 h, 48 h and 72 h. [file 13014_2019_1418_MOESM10_ESM.tif]

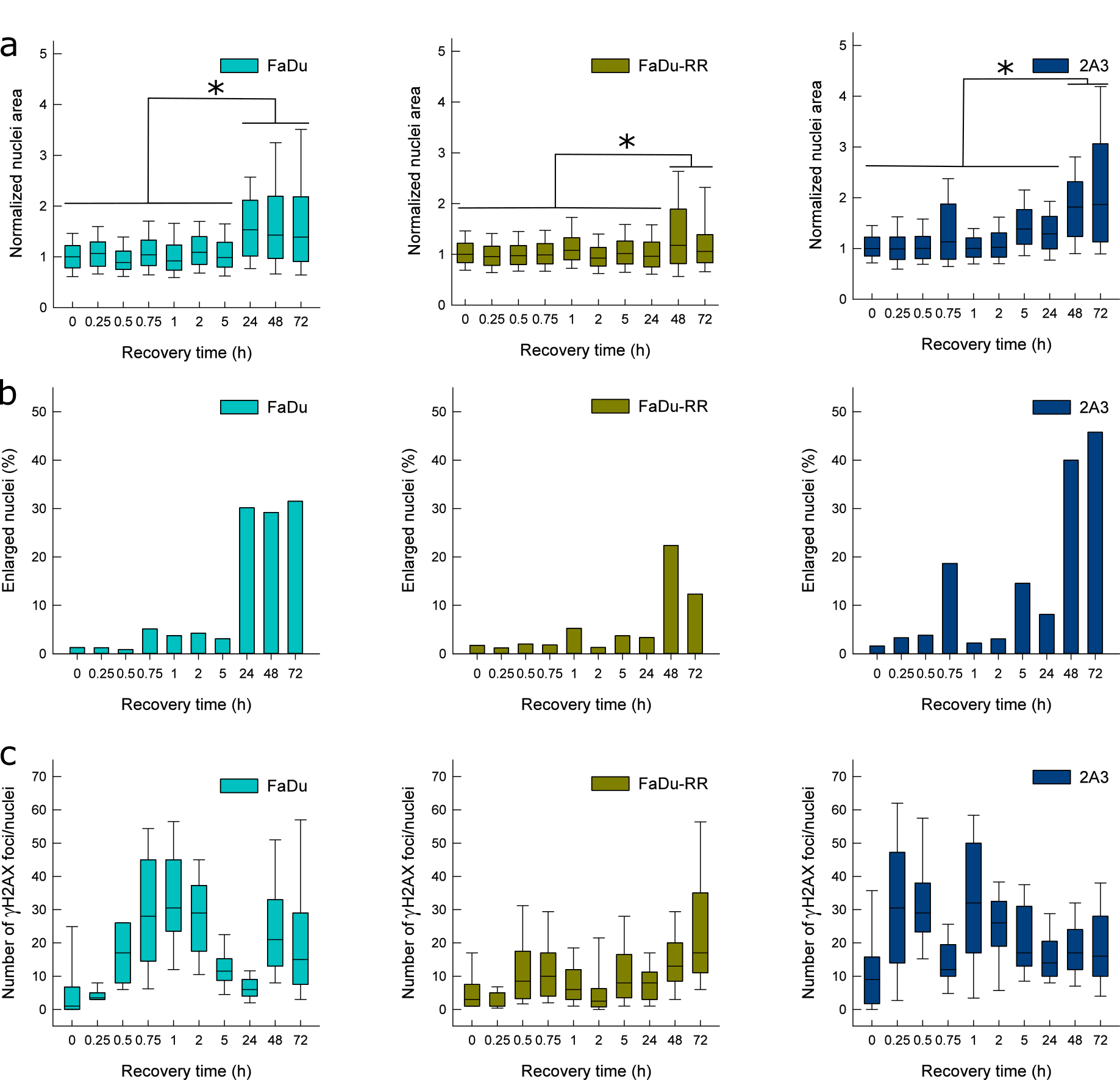

Supplement: Supplementary file 11 — Additional file 11: Figure S6. (a) Normalized nuclei area, (b) percent of enlarged nuclei, and (c) median number of γH2AX foci/nuclei in parental FaDu, radioresistant FaDu-RR, and radiosensitive 2A3 cells at various recovery times after 5 Gy irradiation. Box plots are median with 25th and 75th percentile with 10th and 90th percentiles as bottom and top whiskers. Bars are pooled data from three independent experiments. * indicates significantly enlarged normalized nuclei area compared to other groups. [file 13014_2019_1418_MOESM11_ESM.tif]

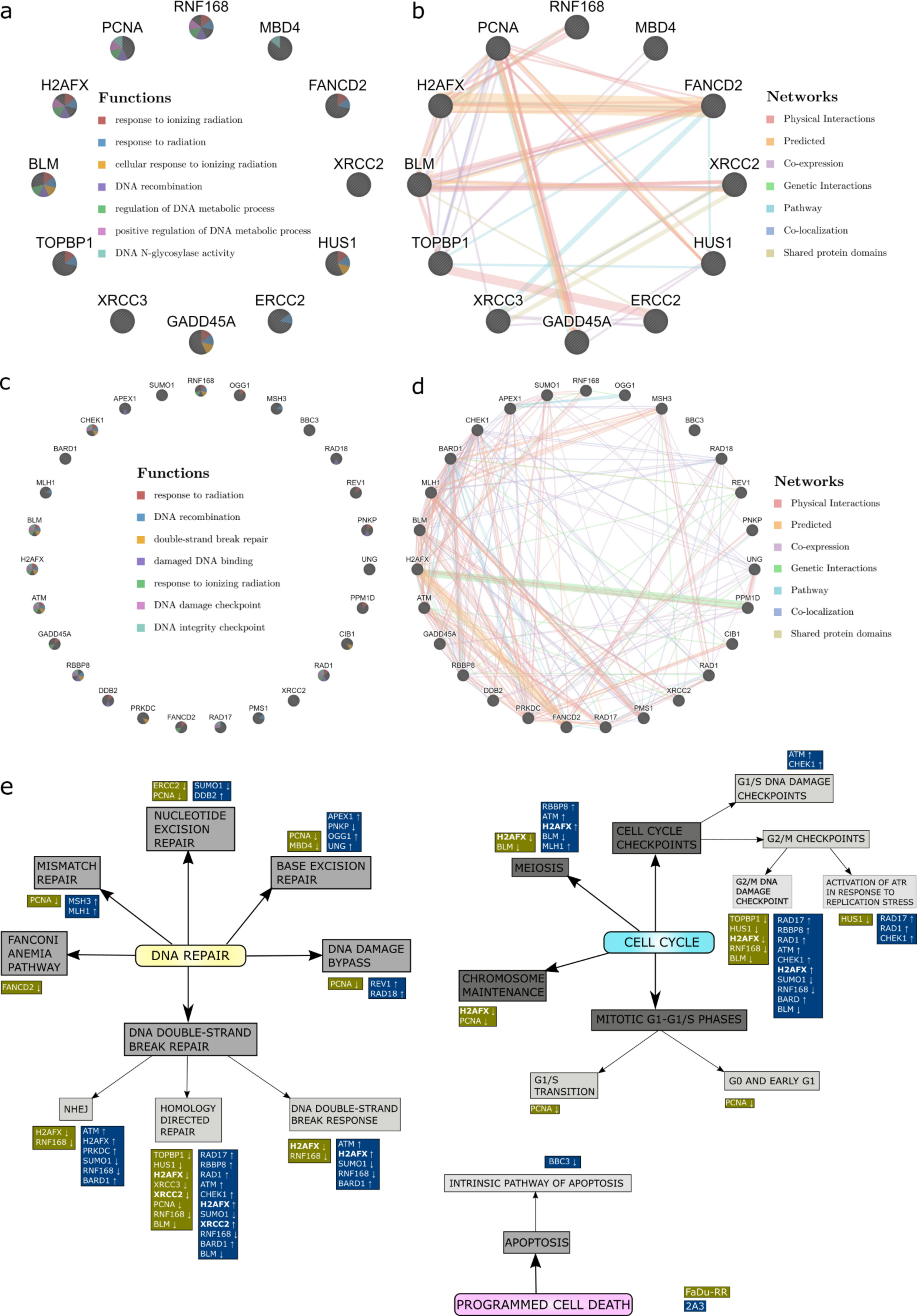

Supplement: Supplementary file 12 — Additional file 12: Figure S7. Functional analysis of DNA damage signalling and repair gene expression in non-irradiated isogenic cells. (a) Top 7 functions associated with the differentially expressed genes in radioresistant FaDu-RR cells. (b) Interactions between the differentially expressed genes in radioresistant FaDu-RR cells. (c) Top 7 functions associated with the differentially expressed genes in radiosensitive 2A3 cells. (d) Interactions between the differentially expressed genes in radiosensitive 2A3 cells. Functions of and interactions between the genes were visualized through GeneMANIA. Circles represent differentially expressed genes in comparison to non-irradiated parental FaDu cells. The colours within the circle represent specific functions associated with the specific gene. The coloured links between the genes represent the type of interaction between the differentially expressed genes. (e) Schematic diagram of differentially expressed genes involved in specific over-represented pathways of DNA repair, cell cycle and programmed cell death in non-irradiated radioresistant FaDu-RR (green box) and radiosensitive 2A3 cells (blue box). Genes in bold are differentially expressed in both radioresistant FaDu-RR and radiosensitive 2A3 cells. An upward pointing arrow indicates over-expression, a downward pointing arrow indicates under-expression of a specific gene. A schematic diagram is adapted from Reactome Pathway Database. [file 13014_2019_1418_MOESM12_ESM.pdf]

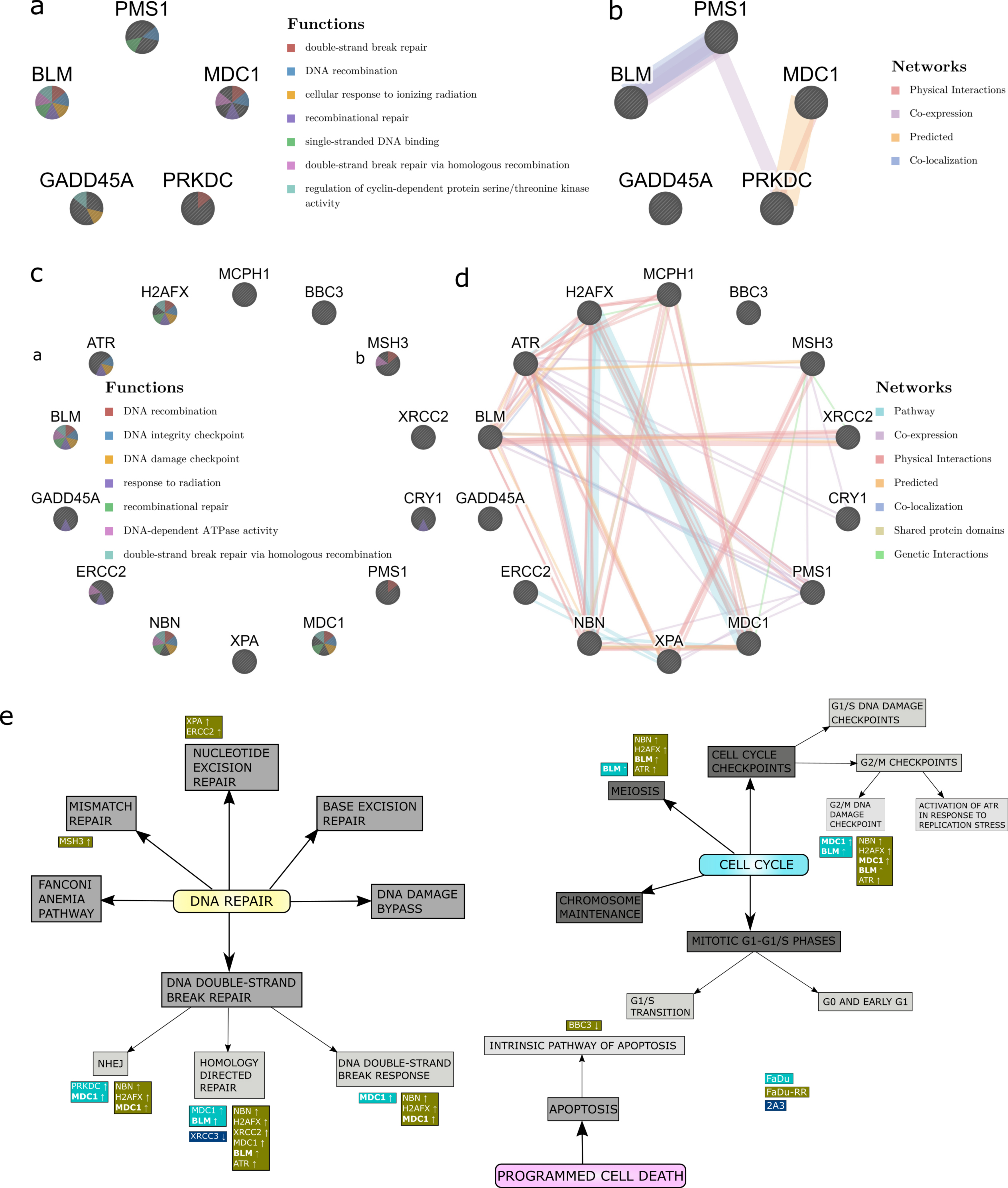

Supplement: Supplementary file 13 — Additional file 13: Figure S8. Functional analysis of DNA damage signalling and repair gene expression in irradiated isogenic cells. (a) Top 7 functions associated with the differentially expressed genes in 5 Gy-irradiated parental FaDu cells. (b) Interactions between the differentially expressed genes in 5 Gy-irradiated parental FaDu cells. (c) Top 7 functions associated with the differentially expressed genes in 5 Gy-irradiated radioresistant FaDu-RR cells. (d) Interactions between the differentially expressed genes in 5 Gy-irradiated radioresistant FaDu-RR cells. Functions of and interactions between the genes were visualized through GeneMANIA. Circles represent differentially expressed genes in 5 Gy-irradiated cells relative to pertinent non-irradiated cells. The colors within the circle represent specific functions associated with the specific gene. The colored links between the genes represent the type of interaction between the differentially expressed genes. (e) Schematic diagram of differentially expressed genes involved in specific pathways of DNA repair, cell cycle and programmed cell death in 5 Gy-irradiated parental FaDu (light blue box), radioresistant FaDu-RR (green box) and radiosensitive 2A3 cells (blue box). Genes in bold are differentially expressed in both parental FaDu and radioresistant FaDu-RR cells. An upward pointing arrow indicates over-expression; a downward pointing arrow indicates under-expression of a specific gene. A schematic diagram is adapted from Reactome Pathway Database. [file 13014_2019_1418_MOESM13_ESM.pdf]

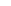

■ DNA recombination

■ Shared protein domains

- Co-localization

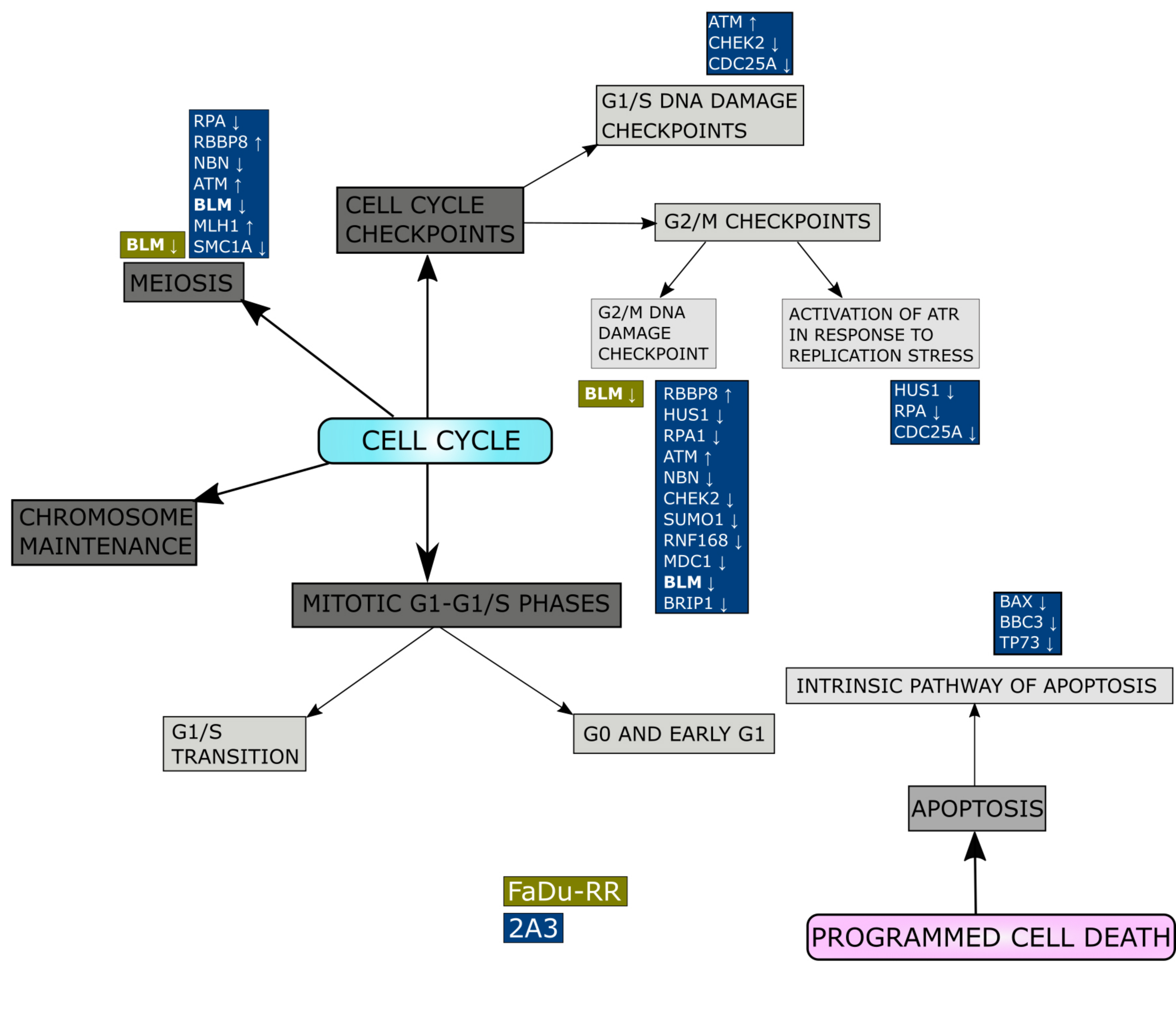

Supplement: Supplementary file 14 — Additional file 14: Figure S9. Functional analysis of DNA damage signalling and repair gene expression in irradiated isogenic cells. (a) Top 7 functions associated with the differentially expressed genes in 5 Gy-irradiated radioresistant FaDu-RR cells. (b) Interactions between the differentially expressed genes in 5 Gy-irradiated radioresistant FaDu-RR cells. (c) Top 7 functions associated with the differentially expressed genes in 5 Gy-irradiated radiosensitive 2A3 cells. (d) Interactions between the differentially expressed genes in 5 Gy-irradiated radiosensitive 2A3 cells. Functions of and interactions between the genes were visualized through GeneMANIA. Circles represent differentially expressed genes in comparison to 5 Gy-irradiated parental FaDu cells. Colours within the circle represent specific functions associated with the specific gene. The coloured links between the genes represent the type of interaction between the differentially expressed genes. (e) Schematic diagram of differentially expressed genes involved in specific pathways of DNA repair, cell cycle and programmed cell death in 5 Gy-irradiated radioresistant FaDu-RR (green box) and radiosensitive 2A3 cells (blue box). Genes in bold are differentially expressed in both radioresistant FaDu-RR and radiosensitive 2A3 cells. An upward pointing arrow indicates over-expression, a downward pointing arrow indicates under-expression of a specific gene. A schematic diagram is adapted from Reactome Pathway Database. [file 13014_2019_1418_MOESM14_ESM.pdf]
