# Supplementary material for: Mechanisms of different response to ionizing irradiation in isogenic head and neck cancer cell lines
Source: Radiat Oncol. 2019 Nov 27;14:214. doi: 10.1186/s13014-019-1418-6 (PMC6882348; doi:10.1186/s13014-019-1418-6)
Supplement: Supplementary file 5 — Additional file 5: Table S1. A list of genes included on RT2 Profiler™ PCR Array Human DNA Damage Signaling Pathway (PAHS-029Z, Qiagen). [file 13014_2019_1418_MOESM5_ESM.pdf]

Table S1. A list of genes included on RT^2 Profiler^{TM} PCR Array Human DNA Damage Signaling Pathway (PAHS-029Z, Qiagen).

Unigene	Refseq	Symbol	Description	RT2 Catalog
Hs.431048	NM_005157	*ABL1*	C-abl oncogene 1, non-receptor tyrosine kinase	PPH00087E
Hs.73722	NM_080649	*APEX1*	APEX nuclease (multifunctional DNA repair enzyme) 1	PPH02201A
Hs.367437	NM_000051	*ATM*	Ataxia telangiectasia mutated	PPH00325C
Hs.271791	NM_001184	*ATR*	Ataxia telangiectasia and Rad3 related	PPH01318B
Hs.694840	NM_032166	*ATRIP*	ATR interacting protein	PPH66805A
Hs.533526	NM_000489	*ATRX*	Alpha thalassemia/mental retardation syndrome X-linked	PPH00470F
Hs.591642	NM_000465	*BARD1*	BRCA1 associated RING domain 1	PPH09451A
Hs.624291	NM_004324	*BAX*	BCL2-associated X protein	PPH00078B
Hs.467020	NM_014417	*BBC3*	BCL2 binding component 3	PPH02204C
Hs.725208	NM_000057	*BLM*	Bloom syndrome, RecQ helicase-like	PPH02711B
Hs.194143	NM_007294	*BRCA1*	Breast cancer 1, early onset	PPH00322F
Hs.128903	NM_032043	*BRIP1*	BRCA1 interacting protein C-terminal helicase 1	PPH10375A
Hs.437705	NM_001789	*CDC25A*	Cell division cycle 25 homolog A (S. pombe)	PPH00930A
Hs.656	NM_001790	*CDC25C*	Cell division cycle 25 homolog C (S. pombe)	PPH02198F
Hs.184298	NM_001799	*CDK7*	Cyclin-dependent kinase 7	PPH00935E
Hs.370771	NM_000389	*CDKN1A*	Cyclin-dependent kinase inhibitor 1A (p21, Cip1)	PPH00211E
Hs.24529	NM_001274	*CHEK1*	CHK1 checkpoint homolog (S. pombe)	PPH00940C
Hs.291363	NM_007194	*CHEK2*	CHK2 checkpoint homolog (S. pombe)	PPH00921B
Hs.715556	NM_006384	*CIB1*	Calcium and integrin binding 1 (calmyrin)	PPH12320C
Hs.151573	NM_004075	*CRY1*	Cryptochrome 1 (photolyase-like)	PPH06231B
Hs.82201	NM_001896	*CSNK2A2*	Casein kinase 2, alpha prime polypeptide	PPH02197F
Hs.290758	NM_001923	*DDB1*	Damage-specific DNA binding protein 1, 127kDa	PPH01515A
Hs.700338	NM_000107	*DDB2*	Damage-specific DNA binding protein 2, 48kDa	PPH01726A
Hs.505777	NM_004083	*DDIT3*	DNA-damage-inducible transcript 3	PPH00310A
Hs.435981	NM_001983	*ERCC1*	Excision repair cross-complementing rodent repair deficiency, complementation group 1 (includes overlapping antisense sequence)	PPH01539A
Hs.487294	NM_000400	*ERCC2*	Excision repair cross-complementing rodent repair deficiency, complementation group 2	PPH01550C
Hs.498248	NM_130398	*EXO1*	Exonuclease 1	PPH02715A
Hs.744083	NM_000135	*FANCA*	Fanconi anemia, complementation group A	PPH15968E

Hs.208388	NM_033084	*FANCD2*	Fanconi anemia, complementation group D2	PPH14413A
Hs.591084	NM_004629	*FANCG*	Fanconi anemia, complementation group G	PPH20387A
Hs.409065	NM_004111	*FEN1*	Flap structure-specific endonuclease 1	PPH00502B
Hs.80409	NM_001924	*GADD45A*	Growth arrest and DNA-damage-inducible, alpha	PPH00148B
Hs.9701	NM_006705	*GADD45G*	Growth arrest and DNA-damage-inducible, gamma	PPH02207A
Hs.477879	NM_002105	*H2AFX*	H2A histone family, member X	PPH12636B
Hs.152983	NM_004507	*HUS1*	HUS1 checkpoint homolog (S. pombe)	PPH00922B
Hs.1770	NM_000234	*LIG1*	Ligase I, DNA, ATP-dependent	PPH02094A
Hs.432642	NM_002969	*MAPK12*	Mitogen-activated protein kinase 12	PPH01779A
Hs.35947	NM_003925	*MBD4*	Methyl-CpG binding domain protein 4	PPH02709A
Hs.593807	NM_024596	*MCPH1*	Microcephalin 1	PPH14515B
Hs.653495	NM_014641	*MDC1*	Mediator of DNA-damage checkpoint 1	PPH20213B
Hs.195364	NM_000249	*MLH1*	MutL homolog 1, colon cancer, nonpolyposis type 2 (E. coli)	PPH00196F
Hs.436650	NM_014381	*MLH3*	MutL homolog 3 (E. coli)	PPH02700A
Hs.459596	NM_002434	*MPG*	N-methylpurine-DNA glycosylase	PPH02102A
Hs.192649	NM_005590	*MRE11A*	MRE11 meiotic recombination 11 homolog A (S. cerevisiae)	PPH01097C
Hs.597656	NM_000251	*MSH2*	MutS homolog 2, colon cancer, nonpolyposis type 1 (E. coli)	PPH00197E
Hs.648635	NM_002439	*MSH3*	MutS homolog 3 (E. coli)	PPH02195A
Hs.492208	NM_002485	*NBN*	Nibrin	PPH00946C
Hs.66196	NM_002528	*NTHL1*	Nth endonuclease III-like 1 (E. coli)	PPH02720A
Hs.380271	NM_002542	*OGG1*	8-oxoguanine DNA glycosylase	PPH02103A
Hs.177766	NM_001618	*PARP1*	Poly (ADP-ribose) polymerase 1	PPH00686B
Hs.147433	NM_182649	*PCNA*	Proliferating cell nuclear antigen	PPH00216B
Hs.111749	NM_000534	*PMS1*	PMS1 postmeiotic segregation increased 1 (S. cerevisiae)	PPH02155B
Hs.715590	NM_000535	*PMS2*	PMS2 postmeiotic segregation increased 2 (S. cerevisiae)	PPH02095E
Hs.78016	NM_007254	*PNKP*	Polynucleotide kinase 3'-phosphatase	PPH02725A
Hs.286073	NM_003620	*PPM1D*	Protein phosphatase, Mg2+/Mn2+ dependent, 1D	PPH02151A
Hs.631593	NM_014330	*PPP1R15A*	Protein phosphatase 1, regulatory (inhibitor) subunit 15A	PPH02081E
Hs.491682	NM_006904	*PRKDC*	Protein kinase, DNA-activated, catalytic polypeptide	PPH01309C
Hs.38114	NM_002853	*RAD1*	RAD1 homolog (S. pombe)	PPH00741F
Hs.16184	NM_002873	*RAD17*	RAD17 homolog (S. pombe)	PPH00929F
Hs.375684	NM_020165	*RAD18*	RAD18 homolog (S. cerevisiae)	PPH02740A
Hs.81848	NM_006265	*RAD21*	RAD21 homolog (S. pombe)	PPH10216A

Hs.633509	NM_005732	*RAD50*	RAD50 homolog (S. cerevisiae)	PPH00956B
Hs.631709	NM_002875	*RAD51*	RAD51 homolog (S. cerevisiae)	PPH00942F
Hs.172587	NM_133509	*RAD51B*	RAD51 homolog B (S. cerevisiae)	PPH02654B
Hs.655354	NM_004584	*RAD9A*	RAD9 homolog A (S. pombe)	PPH00944C
Hs.546282	NM_002894	*RBBP8*	Retinoblastoma binding protein 8	PPH00954F
Hs.443077	NM_016316	*REV1*	REV1 homolog (S. cerevisiae)	PPH21080A
Hs.250648	NM_152617	*RNF168*	Ring finger protein 168	PPH14759A
Hs.485278	NM_183078	*RNF8*	Ring finger protein 8	PPH08534A
Hs.461925	NM_002945	*RPA1*	Replication protein A1, 70kDa	PPH02730A
Hs.369779	NM_012238	*SIRT1*	Sirtuin 1	PPH02188A
Hs.211602	NM_006306	*SMC1A*	Structural maintenance of chromosomes 1A	PPH14489A
Hs.81424	NM_003352	*SUMO1*	SMT3 suppressor of mif two 3 homolog 1 (S. cerevisiae)	PPH00973F
Hs.593379	NM_007027	*TOPBP1*	Topoisomerase (DNA) II binding protein 1	PPH10470A
Hs.437460	NM_000546	*TP53*	Tumor protein p53	PPH00213F
Hs.440968	NM_005657	*TP53BP1*	Tumor protein p53 binding protein 1	PPH02165A
Hs.192132	NM_005427	*TP73*	Tumor protein p73	PPH00725A
Hs.191334	NM_003362	*UNG*	Uracil-DNA glycosylase	PPH01727E
Hs.654364	NM_000380	*XPA*	Xeroderma pigmentosum, complementation group A	PPH01524C
Hs.475538	NM_004628	*XPC*	Xeroderma pigmentosum, complementation group C	PPH01536F
Hs.98493	NM_006297	*XRCC1*	X-ray repair complementing defective repair in Chinese hamster cells 1	PPH01741A
Hs.647093	NM_005431	*XRCC2*	X-ray repair complementing defective repair in Chinese hamster cells 2	PPH01694A
Hs.592325	NM_005432	*XRCC3*	X-ray repair complementing defective repair in Chinese hamster cells 3	PPH02208A
Hs.292493	NM_001469	*XRCC6*	X-ray repair complementing defective repair in Chinese hamster cells 6	PPH02175A
Hs.520640	NM_001101	*ACTB*	Actin, beta	PPH00073G
Hs.534255	NM_004048	*B2M*	Beta-2-microglobulin	PPH01094E
Hs.592355	NM_002046	*GAPDH*	Glyceraldehyde-3-phosphate dehydrogenase	PPH00150F
Hs.412707	NM_000194	*HPRT1*	Hypoxanthine phosphoribosyltransferase 1	PPH01018C
Hs.546285	NM_001002	*RPLP0*	Ribosomal protein, large, P0	PPH21138F
N/A	SA_00105	HGDC	Human Genomic DNA Contamination	
N/A	SA_00104	RTC	Reverse Transcription Control	PPX63340A
N/A	SA_00104	RTC	Reverse Transcription Control	PPX63340A
N/A	SA_00104	RTC	Reverse Transcription Control	PPX63340A
