# Supplementary material for: Mechanisms of different response to ionizing irradiation in isogenic head and neck cancer cell lines
Source: Radiat Oncol. 2019 Nov 27;14:214. doi: 10.1186/s13014-019-1418-6 (PMC6882348; doi:10.1186/s13014-019-1418-6)
Supplement: Supplementary file 6 — Additional file 6: Table S2. Radiosensitivity parameters describing survival advantage of isogenic head and neck SCC cells with different radiosensitivity. [file 13014_2019_1418_MOESM6_ESM.pdf]

**Table S2.** Radiosensitivity parameters describing survival advantage of isogenic head and neck SCC cells with different radiosensitivity.

| Cells | SF2 [1] | $ED_{50}$ [2] (DMF [3]) | $D_{10}$ [4] (DMF) | α[5] | β [6] | α/β [7] |
|---|---|---|---|---|---|---|
| FaDu | 0.42 ± 0.02 | 1.60 ± 0.11 (/) | 4.15 ± 0.13 (/) | 0.34 ± 0.04 | 0.05 ± 0.004 | 7.8 ± 1.2 |
| FaDu-R1 | 0.59 ± 0.03* | 2.28 ± 0.10 (1.4) | 4.92 ± 0.16 (1.2) | 0.13 ± 0.05* | 0.07 ± 0.01 | 2.6 ± 1.5 |
| FaDu-R2 | 0.60 ± 0.04* | 2.44 ± 0.15* (1.5) | 4.99 ± 0.24 (1.2) | 0.13 ± 0.03* | 0.06 ± 0.008 | 2.1 ± 0.4 |
| FaDu-RR | 0.67 ± 0.02** | 2.57 ± 0.09** (1.6) | 5.34 ± 0.11** (1.3) | 0.06 ± 0.01** | 0.07 ± 0.003 | 0.8 ± 0.2** |
| 2A3 | 0.38 ± 0.09 | 1.17 ± 0.18ǂ (0.7) | 4.07 ± 0.35 (1.0) | 0.51 ± 0.10ǂ | 0.05 ± 0.02 | 20.3 ± 7.8ǂ |

[1] SF2 – surviving fraction at 2 Gy; [2] $ED_{50}$ – effective dose (Gy) killing 50% of the cells; [3] DMF – dose-modifying factor; [4] $D_{10}$ – dose (Gy) required to kill 90% of the cells; [5] α – linear coefficient ($Gy^{-1}$) from the LQ model; [6] β – quadratic coefficient ($Gy^{-2}$) from the linear-quadratic model; [7] α/β – dose (Gy) at which linear contribution to damage equals the quadratic contribution; values are mean ± SEM from at least three independent experiments; * indicates significant difference compared to FaDu cells; ** indicates significant difference compared to FaDu and 2A3 cells; ǂ indicates significant difference compared to FaDu and FaDu-RR cells ($P < 0.05$)
