# Supplementary material for: Mechanisms of different response to ionizing irradiation in isogenic head and neck cancer cell lines
Source: Radiat Oncol. 2019 Nov 27;14:214. doi: 10.1186/s13014-019-1418-6 (PMC6882348; doi:10.1186/s13014-019-1418-6)
Supplement: Supplementary file 8 — Additional file 8: Table S3. Percentage of early and late apoptotic cells in control non-irradiated isogenic cells. [file 13014_2019_1418_MOESM8_ESM.pdf]

Table S3. Percentage of early and late apoptotic cells in control non-irradiated isogenic cells.

	**Early apoptotic cells (% ± SEM)**			**Late apoptotic cells (% ± SEM)**		
Time (h)	FaDu	FaDu-RR	2A3	FaDu	FaDu-RR	2A3
5	3.0 ± 0.6	2.0 ± 0.7	2.0 ± 0.3	6.7 ± 0.9	8.4 ± 2.1*	6.1 ± 0.7
24	2.9 ± 0.4	2.1 ± 0.4	2.0 ± 0.2	4.6 ± 0.2	5.5 ± 0.8	4.7 ± 0.8
48	2.3 ± 0.9	1.5 ± 0.2	2.0 ± 0.3	3.3 ± 0.9	4.0 ± 1.0	4.5 ± 1.0
72	5.9 ± 0.5**	2.1 ± 0.3	2.5 ± 0.3	4.8 ± 0.5	3.2 ± 0.4	4.6 ± 1.0

** significant difference compared to FaDu 5, 24, and 48 h. * significant difference compared to FaDu-RR 72 h. Values are AM ± SEM.
